# Fractal–fractional and stochastic analysis of norovirus transmission epidemic model with vaccination effects

**DOI:** 10.1038/s41598-021-03732-8

**Published:** 2021-12-21

**Authors:** Ting Cui, Peijiang Liu, Anwarud Din

**Affiliations:** 1 School of Economics, Guangdong University of Finance and Economics, Guangzhou, 510320 People’s Republic of China; 2 School of Statistics and Mathematics, Guangdong University of Finance and Economics, Guangzhou, 510320 People’s Republic of China; 3 School of Statistics and Mathematics, Guangdong University of Finance and Economics, Big Data and Educational Statistics Application Laboratory, Guangzhou, 510320 People’s Republic of China; 4 Department of Mathematics, Sun Yat-Sen University, Guangzhou, 510275 People’s Republic of China

**Keywords:** Systems biology, Diseases, Mathematics and computing, Physics

## Abstract

In this paper, we investigate an norovirus (NoV) epidemic model with stochastic perturbation and the new definition of a nonlocal fractal–fractional derivative in the Atangana–Baleanu–Caputo (ABC) sense. First we present some basic properties including equilibria and the basic reproduction number of the model. Further, we analyze that the proposed stochastic system has a unique global positive solution. Next, the sufficient conditions of the extinction and the existence of a stationary probability measure for the disease are established. Furthermore, the fractal–fractional dynamics of the proposed model under Atangana–Baleanu–Caputo (ABC) derivative of fractional order “${p}$” and fractal dimension “${q}$” have also been addressed. Besides, coupling the non-linear functional analysis with fixed point theory, the qualitative analysis of the proposed model has been performed. The numerical simulations are carried out to demonstrate the analytical results. It is believed that this study will comprehensively strengthen the theoretical basis for comprehending the dynamics of the worldwide contagious diseases.

## Introduction

The NoV is disseminated by numerous factors that can promptly surge the transmission and henceforth the subsequent disease. The virus is known to be relatively sporadic, with more than half of the infections occurring during the cold season^1^. Seasonal fluctuation is attributed to ecological factors as well as demographic behaviour. Norovirus, for instance, is more easily feasting in cooler temperatures and might be aided by greater rainfall^2,3^. Other factors in the population may influence the intensity of norovirus epidemics. The virus infects people of all ages, however it is most common in kids below the age of five^4^. According to epidemiological studies, that the very earliest norovirus disease arises in childhood. Serious problems and fatality are often more common among the aging and handicapped, according to^5,6^. As a result, while disease is self-limiting in healthy people, the consequences for certain high-risk groups can be devastating. Such populations have also been observed to have much longer shedding intervals, which could lengthen an epidemic^7–10^.

Scholars from different fields have been working to prevent or minimize the rate of infection in certain groups. Mathematicians too have worked on the evaluation of relevant nonlinear dynamics of problems connected to infection, such as epidemics (see, for example^11–13^). Since 1990, mathematicians and biologists have been working hard to learn more about how epidemics travel and how to prevent them from growing in the community. In the area of preventing diseases, mathematicians are also playing an important contribution by employing mathematical modeling approaches as well as optimal solutions^14–20^. Infection modelling and study have become increasingly popular in current years as a means of better comprehending the mechanisms of developing epidemics. The approaches of mathematical modeling and optimality may then be employed to come up with a decent control strategy or technique for managing infectious diseases. Most academics have highlighted the stability and optimality for viral models with a non-linear incident operator using deterministic model techniques^21,22^. To come up with an effective control strategy or treatment for infectious disease, mathematical modelling in various dynamical systems, such as fractional and stochastic modelling, might be applied.

The integer order calculus of differentiation and integration has been developed to rational or complex numbers in modern calculus, illustrating the predicament among two integer numbers as in. The fractional differential equation is also valuable for gaining both analytical and numerical solutions for a multitude of problems^23,24^. Because establishing a precise solution is tough, numerous researchers look at FDEs for optimising and estimated solutions using pre-existing methodologies. They used Modified Euler approaches, Taylor’s series approach, Adams Bash-Forth techniques, predictor-corrector strategy, and other integral transforms, as well as wave-lets methods, to cope of the problem numerically. Recently, Atangana^25^ introduced a new nonlocal operator with the combination of fractional order and fractal dimension known as fractal–fractional (FF) differential and integral operators. The FF operator has been applied as an effective tool in describing various phenomena in many areas of science and epidemiology to explore the complex real word problems that could not be modeled with classical and fractional differential and integral operators with single order.

Numerous random order derivatives with an extra degree of freedom of choice have been explored using the fractional-order (FO) and integer-order mathematical model. When compared to deterministic models, it covers the entire spectrum to every compartment and a more accurate outcome than the natural order model also FO model, and stochastic differential equations could provide a high degree of stability. Because the findings of each investigation in a stochastic process varies from the others, we must run the models multiple times and look for patterns in the expected outcomes. Several authors have studied the stability analysis of different epidemic schemes including a non-linear incidence mapping for stochastic models^26–28^.

The five stochastic differential equations are used to proposed a stochastic epidemic mathematical system for NoV. The entire population is split in five partitions, each of which represents a sub-population: susceptible (*H*), vaccinated (*V*), asymptomatic or exposed (*U*), symptomatic or infected (*A*), and recovered (*C*), i.e., $H(t)+V(t)+U(t)+A(t)+C(t))=N(t)$ The equations describing the model are

1 $$\begin{aligned} \displaystyle dH(t)&=\bigg [\Lambda -\frac{\eta H(t)A(t)}{N}-(\rho +d) H(t)\bigg ]dt+\zeta _1H(t)dW_1(t),\\ \displaystyle dV(t)&=\bigg [\rho H(t)-\frac{(1-\tau )\eta V(t)A(t)}{N}-d V(t)\bigg ]dt+\zeta _2V(t)dW_2(t),\\ \displaystyle dU(t)&=\bigg [\frac{\eta H(t)A(t)}{N}+\frac{(1-\tau )\eta V(t)A(t)}{N}-(\alpha +d)U(t)\bigg ]dt+\zeta _3UdW_3(t),\\ \displaystyle dA(t)&=\bigg [\alpha U(t)-(\delta +d) A(t)\bigg ]dt+\zeta _4A(t)dW_4(t),\\ \displaystyle dC(t)&=\bigg [\delta A(t)-d C(t)\bigg ]dt+\zeta _5C(t)dW_5(t).\\ \end{aligned}$$

Here $W_1(t), W_2(t), W_3(t), W_4(t), W_5(t)$ are independently standard Brownian motions, and $\zeta _{1}, \zeta _{2}, \zeta _{3}, \zeta _{4}, \zeta _{5}$ are the intensities of standard Gaussain white noises, correspondingly. The parameters description given in Table 2. Here, vaccinated people also become infected via contact with symptomatic people. Note that, $0< \tau < 1$ means $\tau = 1$ perfect vaccine, while $\tau = 0$ represents a vaccine that offers no protection at all.

The rest of the article layout has been established; In “Deterministic state stability” section, we determined the deterministic stability of system (1). We prove the global positive solution for system (1) in “The existence and uniqueness of positive solution” section. We employed a stochastic threshold strategy to decrease the epidemic in “Extinction of the disease” section. And in “Stationary distribution and ergodicity” section we evaluate a stationary distribution of the stochastic model. In “A fractal–fractional NoV model with Mittag–Leffler kernel” section, we analyze the proposed model through Fractal–fractional Atangana–Baleanu operator. In “Parameter estimation” section, the application of the parameter estimation is presented for the proposed model. In “Numerical simulations” section, we give simulation results to support our theoretical results. In the “Conclusion” section, we are give some conclusions.

## Deterministic state stability

The deterministic form of system (1) is governed by the following set of equations

2 $$\begin{aligned} \frac{dH}{dt}&=\Lambda -\frac{\eta H(t)A(t)}{N}-(\rho +d) H(t),\\ \frac{dV}{dt}&=\rho H(t)-\frac{(1-\tau )\eta V(t)A(t)}{N}-d V(t),\\ \frac{dU}{dt}&=\frac{\eta H(t)A(t)}{N}+\frac{(1-\tau )\eta V(t)A(t)}{N}-(\alpha +d)U(t),\\ \frac{dA}{dt}&=\alpha U(t)-(\delta +d) A(t),\\ \frac{dC}{dt}&=\delta A(t)-d C(t).\\ \end{aligned}$$

with initial conditions

$$\begin{aligned} H(0)=H_0\ge 0,~ V(0)=V_0\ge 0,~ U(0)=U_0\ge 0,~ A(0)=A_0\ge 0,~ C(0)=C_0\ge 0. \end{aligned}$$

The model equilibria provide useful information regarding the model trajectory over time. The model (2) posses two type of equilibrium points, the disease-free (DFE) and endemic equilibrium (EE) points.

### Disease-free equilibrium

The disease-free equilibrium $(G_0)$ can be obtained by equating the right side of equations in system (2) to zero as follows:

3 $$\begin{aligned} G_{0}=\left( S_{0}, V_{0}, U_{0}, A_{0}, C_{0}\right) =\left( \frac{\Lambda }{\rho +d}, \frac{\rho \Lambda }{d(\rho +d)}, 0,0,0\right) . \end{aligned}$$

#### Lemma 1

*The DFE* $G_0$ *is locally and globally asymptotically stable if* $R_0^D < 1$, *and unstable if* $R_0^D > 1$.

#### Proof

The Proof of the Lemma is simple so we omit it here.

Using the next-generation matrix technique, we compute the basic reproductive ratio $R_0^D$ using only the two equations corresponding to compartments *U* and *A* classes from system (2).

$$\begin{aligned} {\mathbf {F}}&=\left[ \begin{array}{cc} 0 &{} \frac{\eta \Lambda [d+(1-\tau ) \rho ]}{d(\rho +d)} \\ 0 &{} 0 \end{array}\right] , \\ {\mathbf {V}}&=\left[ \begin{array}{cc} d+\alpha &{} 0 \\ -\alpha &{} d+\delta \end{array}\right] , \\ {\mathbf {V}}^{-1}&=\left[ \begin{array}{cc} \frac{1}{d+\alpha } &{} 0 \\ \frac{\alpha }{(d+\alpha )(d+\delta )} &{} \frac{1}{d+\delta } \end{array}\right] .\end{aligned}$$

The basic reproductive ratio, defined as the spectral radius of the matrix $\mathbf {F V}^{-1}$, is obtained as

$$\begin{aligned} R_{0}^D=\rho \left( \mathbf {F V}^{-1}\right) =\frac{\alpha \eta \Lambda [d+(1-\tau ) \rho ]}{d(\rho +d)(d+\alpha )(d+\delta )}. \end{aligned}$$

### Endemic equilibrium

The system (2) is also have EE equilibrium point which denoted by $G_1=(H_1,V_1,U_1.A_1,C_1)$, in same way we can find $G_1$, where

$$\begin{aligned} S_{1}&=\frac{\Lambda }{\eta U_{1}+\rho +d}, \\ V_{1}&=\frac{\rho \Lambda }{\left( \eta U_{1}+\rho +d\right) \left[ (1-\tau ) \eta U_{1}+d\right] }, \\ U_{1}&=\frac{\delta +d}{\alpha } U_{1}, \\ A_{1}&=\frac{-B_{2}+\sqrt{B_{2}^{2}-4 B_{1} B_{3}}}{2 B_{1}}, \\ C_{1}&=\frac{\delta }{d} U_{1}, \end{aligned}$$

where

$$\begin{aligned} B_{1}&=\eta ^{2}(1-\tau ), \\ B_{2}&=\eta d\left( 1+\frac{(1-\tau )}{d+(1-\tau ) \rho }\left( 1-R_{0}^D+(1-\tau ) \frac{\rho }{d}\right) \right) , \\ B_{3}&=d(\rho +d)\left( 1-R_{0}^D\right) . \end{aligned}$$

#### Lemma 2

*The EE* $G_1$ *is locally and globally asymptotically stable if* $R_0^D < 1$ *and unstable if* $R_0^D >1$.

#### Proof

The Proof of the Lemma is simple so we omit it here. $\square$

## The existence and uniqueness of positive solution

In order to addresses the existence and uniqueness of the stochastic model (1), we present the following theorem.

### Theorem 1

*The solution of the constructed stochastic epidemiological system* (1) (*H*(*t*), *V*(*t*), *U*(*t*), *A*(*t*), *C*(*t*)) *is unique for* $t\ge 0$ *with initial condition* $(H(0), V(0), U(0), A(0), C(0))\in {\mathbb {C}}^{5}_{+}$. *Additionally, the solution will almost certainly remain in* ${\mathbb {C}}^{5}_{+}$ *with the unit probability, that is*, $(H(0), V(0), U(0), A(0), C(0))\in {\mathbb {C}}^{5}_{+}~\forall ~t\ge 0$ *almost surely (a.s)*.

### Proof

The coefficients used in equations for the initial value of the state variables $(H(t), V(t), U(t), A(t), C(t))\in {\mathbb {C}}^{5}_{+}$ are continuous and locally lipschitz. As a result, there should be a local unique solution of the system

(*H*(*t*), *V*(*t*), *U*(*t*), *A*(*t*), *C*(*t*)) throughout $t\in [0,\tau _e)$. The citations^22,26^ provide a detailed examination of the explosion duration $\tau _e$. To demonstrate the solution’s global character, we must establish that $\tau _e=\infty$ a.s. Suppose we have a comparatively big nonnegative number $k_0$ such that each of the state’s starting conditions are contained within the range $[\frac{1}{k_0},k_0]$. Let the final time be specified as $k\ge k_0$ to every positive integer.

4 $$\begin{aligned} \tau _k=inf\left\{ t\in [0,\tau _e):min\{H(t), V(t), U(t), A(t), C(t)\}\le \frac{1}{k}~\text {or}~max\{H(t), V(t), U(t), A(t), C(t)\}\ge k\right\} . \end{aligned}$$

We will use $\inf \phi =\infty$ throughout this article, whereas $\phi$ stands for the null set. The concept of $\tau _k$ forces us to state that it rises as *k* reaches $\infty$. Replacing $\tau _\infty =\lim _{k\rightarrow \infty }$ for $\tau _e\ge \tau _\infty$ a.s. After demonstrating that $\tau _\infty =\infty$ a.s., we will assert that $\tau _e=\infty$, and therefore (*H*(*t*), *V*(*t*), *U*(*t*), *A*(*t*), *C*(*t*)) will be found in ${\mathbb {C}}^{5}_{+}$ a.s. $\forall t\ge 0$. Hence, proving that $\tau _e=\infty$ a.s. suffices. Instead, two positive constants values $\epsilon$ from (0, 1) and *T* must exist, such that

5 $$\begin{aligned} P\{T\ge \tau _\infty \}>\epsilon . \end{aligned}$$

As a result, the integer $k_1\ge k_0$ exists in the following form

$$\begin{aligned} P\{T\ge \tau _k\}\ge \epsilon ,~\forall ~ k_1\le k. \end{aligned}$$

After that, we’ll look at how to interpret a $C^{2}$-function $H:{\mathbb {C}}^5_{+}\rightarrow {\mathbb {C}}_{+}$ in such a way that

6 $$\begin{aligned} H(H,V,U,A,C)=H+U+V+A+C-5-(\log H+\log V+\log U + \log A+\log C). \end{aligned}$$

It is to be noted that the *H* is a nonnegative function, and it can be verified from the fact that $0\le y-\text {logy}-1,~\forall ~0<y$. Assume that $k_0\le K$ and $0<T$ are arbitrary. Upon applying $At{\hat{o}}'s$ formula to Eq. (6) gives us

7 $$ \begin{aligned} dH(H,V,U,A,C)&=LH(H,V,U,A,C)+\zeta _1(H-1)dW_1(t)+\zeta _2(V-1)dW_2(t)\\&\quad +\zeta _3(U-1)dW_3(t)+\zeta _4(A-1)dW_4(t)+\zeta _5(C-1)dW_5(t). \end{aligned}$$

In Eq. (7), $LH:{\mathbb {C}}^5_{+}\rightarrow {\mathbb {C}}_{+}$ is defined by the following equation

8 $$ \begin{aligned} LH&=\bigg (1-\frac{1}{H}\bigg )\bigg (\Lambda -\frac{\eta HA}{N}-(\rho +d) H\bigg )+\frac{\zeta _1^2}{2} +\bigg (1-\frac{1}{V}\bigg )\bigg (\rho H-\frac{(1-\tau )\eta VA}{N}-d V\bigg )+\frac{\zeta _2^2}{2}\\&\quad+\bigg (1-\frac{1}{U}\bigg )\bigg (\frac{\eta HA}{N}+\frac{(1-\tau )\eta VA}{N}-(\alpha +d)U\bigg )+\frac{\zeta _3^2}{2}+\bigg (1-\frac{1}{A}\bigg )\bigg (\alpha U-(\delta +d) A\bigg )+\frac{\zeta _4^2}{2} \\&\quad+\bigg (1-\frac{1}{C}\bigg )\bigg (\delta A-d C\bigg )+\frac{\zeta _5^2}{2}.\\ \end{aligned}$$

$$\begin{aligned} LH(H,V,U,A,C)&=\Lambda -d(H+V+U+A+C)-\frac{\Lambda }{H}+\frac{\eta A}{N}-\frac{\rho H}{V}-(1-\tau )\eta A-\frac{\eta HA}{NU}-\frac{(1-\tau )\eta VA}{NU}\\&\quad-\frac{\alpha U}{A}-\frac{\delta A}{C}+\rho +5d+\delta +\frac{\zeta _{1}{^2}+\zeta _{2}{^2}+\zeta _{3}{^2}+\zeta _{4}{^2}+\zeta _{5}{^2}}{2}\\&\le \Lambda +\alpha +5d+\rho +\delta +\frac{\zeta _{1}{^2}+\zeta _{2}{^2}+\zeta _{3}{^2}+\zeta _{4}{^2}+\zeta _{5}{^2}}{2}:=K.\\ \end{aligned}$$

Thus,

9 $$\begin{aligned} &{\mathbb {U}}\bigg [H(H(\tau _k\wedge T),V(\tau _k\wedge ,U(\tau _k\wedge T),A(\tau _k\wedge T),C(\tau _k\wedge T))\bigg ]\\&\quad \le H(H(0),V(0),U(0),A(0),C(0))+{\mathbb {U}}\bigg [\int ^{\tau _k\wedge T}_0Kdt\bigg ],\\&\quad \le H(H(0),V(0),U(0),A(0),C(0))+KT. \end{aligned}$$

Setting $\Omega _k=\{\tau _k\le T\}$ for $k\ge k_1$ and by Eq. (5), $P(\Omega _k)\ge \epsilon$. Note that for each $\omega$ from $\Omega _k$ there must exist one or more than one $H(\tau _k,\omega )$, $V(\tau _k,\omega )$, $U(\tau _k,\omega )$, $A(\tau _k,\omega )$ and $C(\tau _k,\omega )$ which equals

$\frac{1}{k}$ or *k*. As a result $H(H(\tau _k),V(\tau _k),U(\tau _k),A(\tau _k),C(\tau _k))$ is no less then $\frac{1}{k}-1+\text {log}k$ or $k-1-\text {log}k$. Therefore,

10 $$\begin{aligned} H(H(\tau _k), V(\tau _k), U(\tau _k), A(\tau _k), C(\tau _k))\ge \bigg (\frac{1}{k}-1+\text {log}k\bigg )\wedge {\mathbb {U}}\big (k-1-\text {log}k\big ). \end{aligned}$$

By using Eqs. (5) and (9), we can write

11 $$\begin{aligned} H(H(0),V(0),U(0),A(0),C(0))+KT&\ge {\mathbb {U}}\bigg [1_{\Omega (\omega )}H\big (H(\tau _k),V(\tau _k),U(\tau _k,A(\tau _k),C(\tau _k)\big )\bigg ]\\&\ge \epsilon \bigg [\bigg (\frac{1}{k}-1+\text {log}k\bigg )\wedge (k-1-\text {log}k)\bigg ]. \end{aligned}$$

The indicator function of $\Omega$ is represented as $1_{\Omega (\omega )}$. As we get closer to $\infty$, the contradiction $\infty >H\big (H(0),V(0),U(0),A(0),C(0))\big )+MT=\infty$ emerges, indicating that $\tau _\infty =\infty$ a.s. $\square$

## Extinction of the disease

This section focuses on the criteria for disease’s extinction in system (1). Prior to prove the major findings, let’s look at an important lemmas.

Let

12 $$\begin{aligned} \big <X(t)\big >=\frac{1}{t}\int _0^t x(r)dr. \end{aligned}$$

### Lemma 3

^13,14^   (Strong Law of Large Number) *If* $M=\{M\}_{t\ge 0}$ *is now a continuous and real-valued local martingale that vanishes at* $t=0$,

13 $$\begin{aligned}&\lim _{t\rightarrow \infty }\big<M,M\big>_t=\infty , ~\text {a.s.,~implies that} \lim _{t\rightarrow \infty }\frac{\big<M_t\big>}{\big<M,M\big>_t}=0,~\text {a.s.},~\text {and also}\\&\quad \lim _{t\rightarrow \infty }sup\frac{\big<M,M\big>_t}{t}<0,~\text {a.s., implies that}~\lim _{t\rightarrow \infty }\frac{\big <M_t\big >}{t}=0,~\text {a.s.} \end{aligned}$$

### Lemma 4

*For arbitrary given starting value* $(H(0), V(0), U(0),A(0), C(0)) \in {\mathbb {C}}_+^5,$ *the solution* (*H*(*t*), *V*(*t*), *U*(*t*), *A*(*t*), *C*(*t*)) *for the system 2 has the upcoming properties:*

14 $$\begin{aligned} \lim _{t\rightarrow \infty }\frac{H(t)}{t}&=0,\\ \lim _{t\rightarrow \infty }\frac{V(t)}{t}&=0,\\ \lim _{t\rightarrow \infty }\frac{U(t)}{t}&=0,\\ \lim _{t\rightarrow \infty }\frac{A(t)}{t}&=0,\\ \lim _{t\rightarrow \infty }\frac{C(t)}{t}&=0~~~~a.s. \end{aligned}$$

*Furthermore, when* $d>\frac{1}{2}(\zeta _1^2\vee \zeta _2^2\vee \zeta _3^2\vee \zeta _4^2\vee \zeta _5^2)$ *holds, then*

15 $$\begin{aligned} \lim _{t\rightarrow \infty }\frac{1}{t}\int _0^tH(r)dW_1(r)&=0,\\ \lim _{t\rightarrow \infty }\frac{1}{t}\int _0^tV(r)dW_2(r)&=0,\\ \lim _{t\rightarrow \infty }\frac{1}{t}\int _0^tU(r)dW_3(r)&=0,\\ \lim _{t\rightarrow \infty }\frac{1}{t}\int _0^tA(r)dW_4(r)&=0,\\ \lim _{t\rightarrow \infty }\frac{1}{t}\int _0^tC(r)dW_5(r)&=0~~~~a.s. \end{aligned}$$

### Proof

We exclude Lemma  4 proof because it is same in Lemma 4.1 in^28^. $\square$

Defined a parameter

16 $$\begin{aligned} R_0= \frac{\alpha \eta 2(\alpha +d)^2}{(\alpha +d)(\delta +d+\frac{\zeta _4^2}{2})(\alpha +d)^2 \wedge (\alpha ^2 \frac{\zeta _3^2}{2})} \end{aligned}$$

### Theorem 2

*If* $R_0<1$ *and* $d >\frac{\zeta _1^2\vee \zeta _2^2\vee \zeta _3^2\vee \zeta _4^2\vee \zeta _5^2}{2},$ *then the root of system* (1) *fulfilling, so given as*:

17 $$\begin{aligned} \lim _{t\rightarrow \infty } \frac{\ln [\alpha U(t)+(\alpha +d)A(t)]}{t}&\le \frac{(\delta +d+\frac{\zeta _4^2}{2})(\alpha +d)^2 \wedge (\alpha ^2 \frac{\zeta _3^2}{2})}{2(\alpha +d)^2}(R_0-1)<0.\\ \end{aligned}$$

### Proof

Describe a differentiable function $G_0$ as

18 $$\begin{aligned} G_0=\ln [\alpha U(t)+(\alpha +d)A(t)]. \end{aligned}$$

Considering Ito’s formula along with using system (1), we get

19 $$ \begin{aligned} dG_0&=\bigg \{ \frac{(\alpha \eta HA)\backslash N-(\alpha +d)(\delta +d)A}{[\alpha U+(\alpha +d)A]}-\frac{\alpha (1-\tau )\eta VA\backslash N}{[\alpha U+(\alpha +d)A]}-\frac{\alpha ^2 \zeta _3^2 U^2+(\alpha +d)\zeta _4^2 A^2}{2([\alpha U+(\alpha +d)A])^2} \bigg \}dt \\&\quad +\frac{\alpha \zeta _3 U}{[\alpha U+(\alpha +d)A]}d W_3+\frac{(\alpha +d) \zeta _4 A}{[\alpha U+(\alpha +d)A]}d W_4\\&\le \bigg \{\frac{\alpha \eta }{(\alpha +d)}-\frac{(\delta +d+\frac{\zeta _4^2}{2})(\alpha +d)^2 A^2+(\alpha ^2 \frac{\zeta _3^2}{2}U^2)}{[\alpha U+(\alpha +d)A]^2}\bigg \}dt+\frac{\alpha \zeta _3 U}{\alpha U+(\alpha +d)A}d W_3+\frac{(\alpha +d) \zeta _4 A}{\alpha U+(\alpha +d)A}d W_4\\&=\bigg \{\frac{\alpha \eta }{(\alpha +d)}-\frac{(\delta +d+\frac{\zeta _4^2}{2})(\alpha +d)^2 \wedge (\alpha ^2 \frac{\zeta _3^2}{2})}{2(\alpha +d)^2}\bigg \}dt+\frac{\alpha \zeta _3 U}{[\alpha U+(\alpha +d)A]}d W_3+\frac{(\alpha +d) \zeta _4 A}{[\alpha U+(\alpha +d)A]}d W_4.\\ \end{aligned}$$

On the both sides of (19), we have integrating with limits from 0 to *t* and dividing with *t* we have the following

20 $$ \begin{aligned} \frac{\ln [\alpha U(t)+(\alpha +d)A(t)]}{t}&\le \frac{\alpha \eta }{(\alpha +d)}-\frac{(\delta +d+\frac{\zeta _4^2}{2})(\alpha +d)^2 \wedge (\alpha ^2 \frac{\zeta _3^2}{2})}{2(\alpha +d)^2}\frac{\ln [\alpha U(0)+(\alpha +d)A(0)]}{t}\\&\quad +\frac{\alpha \zeta _3}{t}\int _0^t \frac{U(r)}{[\alpha U(r)+(\alpha +d)A(r)]}d W_3\\&\quad +\frac{(\alpha +d)\zeta _4}{t}\int _0^t \frac{A(r)}{[\alpha U(r)+(\alpha +d)A(r)]}d W_4 \end{aligned}$$

We’ve used Lemma 3 to get the following

21 $$\begin{aligned} \lim _{t\rightarrow \infty } \frac{\ln [\alpha U(t)+(\alpha +d)A(t)]}{t}&\le \frac{\alpha \eta }{(\alpha +d)}-\frac{(\delta +d+\frac{\zeta _4^2}{2})(\alpha +d)^2 \wedge (\alpha ^2 \frac{\zeta _3^2}{2})}{2(\alpha +d)^2}<0,\\&\le \frac{(\delta +d+\frac{\zeta _4^2}{2})(\alpha +d)^2 \wedge (\alpha ^2 \frac{\zeta _3^2}{2})}{2(\alpha +d)^2}(R_0-1)<0,~~~a.s.\\ \end{aligned}$$

Which demonstrates

22 $$ \begin{aligned} \lim _{t\rightarrow \infty }\left\langle U(t)\right\rangle&=0,\\ \lim _{t\rightarrow \infty }\left\langle A(t)\right\rangle&=0~~~a.s. \end{aligned}$$

It is simple to deduce below from the fourth equation of system (1).

23 $$\begin{aligned} \lim \limits _{t \rightarrow \infty }\left\langle C(t)\right\rangle =0~~~a.s. \end{aligned}$$

Furthermore, on both hand sides of the first equation in system (1), integrating from 0 to *t* and dividing with *t* obtains

24 $$\begin{aligned} \frac{H(t)-H(0)}{t}=\Lambda -\eta \left\langle \frac{HA}{N}\right\rangle -(\rho +d)\left\langle H \right\rangle +\frac{\zeta _1}{t}\int _0^t H(r)dW_1(r), \end{aligned}$$

and considering (22), and Lemma 3, it then follows that

25 $$\begin{aligned} \lim \limits _{t \rightarrow \infty }\left\langle H \right\rangle =\frac{\Lambda }{\rho +d}=H_0~~~a.s. \end{aligned}$$

Similarly, we also can get

26 $$\begin{aligned} \lim \limits _{t \rightarrow \infty }\left\langle V \right\rangle =\frac{\rho \Lambda }{d(\rho +d)}=V_0~~~a.s. \end{aligned}$$

The proof for Theorem 2 is finished. $\square$

## Stationary distribution and ergodicity

When it comes to stochastic systems, there are no endemic equilibria. As a result, the stability analysis could be utilised to investigate the disease’s persistence. As a consequence, one should focus on the existence and uniqueness theory for the stationary distribution, which, in some ways, will help with disease persistence. We shall use Hasminskii’s renowned finding^29^ for this task.

Let *X*(*t*) be a regular Markov process (time-homogeneous) in $C^n_+$ for which the dynamics is as below:

$$\begin{aligned} dX(t) = b(X)dt+\sum ^k_r \zeta _r dW_r(t). \end{aligned}$$

The diffusion matrix is of the form

$$\begin{aligned} A(X) = [a_{ij}(x)],~~a_{ij}(x) = \sum ^k_{r=1}\zeta ^i_r(x)\zeta ^r_j(x). \end{aligned}$$

### Lemma 5

^22,26^ *The stationary distribution of the process* *X*(*t*) *is unique. If there exists a bounded domain having a regular* boundary such that $U,{\bar{U}}\in C^d$ ${\bar{U}}$ *closure* ${\bar{U}}\in C^d,$ *with below properties *

1. *The lowest eigenvalue for A(t) is bounded away form the origin for the open domain U along with its neighbourhood.*
2. *for* $x \in C^d U,$, *the mean time* $\tau$ *(with which a path originating from x reaches the set U) is bounded, and for all compact subset* $K\subset C^n$, $Sup_{x\in k} U^x\tau < \infty$. *When f(.) is an integrable function having measure* $\pi$, *thus*

$$\begin{aligned} P \big (\lim _{T \rightarrow \infty } \frac{1}{T} \int ^T_0 f (X_x(t))dt = \int _{C^d} f(x)\pi (dx)\big ) = 1 \end{aligned}$$

*for each* $$x \in C^d.$$

Describe a parameter

27 $$\begin{aligned} R^s_0 =\frac{d \eta \alpha }{\bigg (\rho +d+\frac{\zeta _{2}{^2}}{2}\bigg )\bigg (\alpha +d+\frac{\zeta _{3}{^2}}{2}\bigg )\bigg (\delta +d+\frac{\zeta _{4}{^2}}{2}\bigg )}. \end{aligned}$$

### Theorem 3

*The system* (1) *solution* (*H*(*t*), *V*(*t*), *U*(*t*), *A*(*t*), *C*(*t*)) *is ergodic, and having a unique stationary distribution. Since* $R^s_0 > 1$, $\pi (.)$ *is used*.

### Proof

To check the condition (2) of the Lemma 5, we should define a non-negative $C^2-$function $V:C^5_+ \rightarrow C_+.$ For which we need to define

$$\begin{aligned} V_1 = H+V+U+A+C-c_1\ln H-c_2\ln U-c_3\ln A, \end{aligned}$$

here the positive constants $c_1, c_2$ and $c_3$ must be calculated afterwards. We get the following results by utilising Itô’s formula and the suggested system (1).

28 $$\begin{aligned} {\mathcal {L}}(H+V+U+A+C)=&\pi -d(H(t)+V(t)+U(t)+A(t)+C(t)),\\ {\mathcal {L}}(-\ln H)=&-\frac{\Lambda }{H}+\frac{\eta A}{N}+(\rho +d)+\frac{\zeta _1^2}{2}, \\ {\mathcal {L}}(-\ln V)=&-\frac{\rho H}{V}+\frac{(1-\tau )\eta A}{N}+d+\frac{\zeta _2^2}{2}, \\ {\mathcal {L}}(-\ln U)=&-\frac{\eta HA}{NU}-\frac{(1-\tau )\eta VA}{NU}+(\alpha +d)+\frac{\zeta _3^2}{2}, \\ {\mathcal {L}}(-\ln A)=&-\frac{\alpha U}{A}+(\delta +d)+\frac{\zeta _4^2}{2}.\\ {\mathcal {L}}(-\ln C)=&-\frac{\delta A}{C}+d+\frac{\zeta _5^2}{2}. \end{aligned}$$

Therefore, we have

$$ \begin{aligned} {\mathcal {L}}V_1&=-d(H(t)+V(t)+U(t)+A(t)+C(t))-\frac{c_1\Lambda }{H}+\frac{c_1\eta A}{N}+c_1\bigg (\rho +d+\frac{\zeta _1^2}{2}\bigg )-\frac{c_2\eta HA}{NU}\\&-\frac{c_2(1-\tau )\eta VA}{NU}+c_2\bigg (\alpha +d+\frac{\zeta _3^2}{2}\bigg )-\frac{c_3\alpha U}{A}+\Lambda +c_3\bigg (\delta +d+\frac{\zeta _4^2}{2}\bigg ). \end{aligned}$$

The above implies that

$$\begin{aligned} {\mathcal {L}}V_1\le&-4\bigg [d(H(t)+V(t)+U(t)+A(t)+C(t))\times \frac{c_1\Lambda }{H}\times \frac{c_2\eta HA}{(H(t)+V(t)+U(t)+A(t)+C(t))U}\times \frac{\alpha U}{A}\bigg ]^\frac{1}{4}\\&\quad +c_1\bigg (\rho +d+\frac{\zeta _1^2}{2}) +c_2\bigg (\alpha +d+\frac{\zeta _3^2}{2}\bigg ) +c_3\bigg (\delta +d+\frac{\zeta _4^2}{2}\bigg )+c_1\frac{\eta A }{N}+\Lambda . \end{aligned}$$

Let

$$\begin{aligned} c_1\bigg (\rho +d+\frac{\zeta _1^2}{2}\bigg )=c_2\bigg (\alpha +d+\frac{\zeta _2^3}{2}\bigg )=c_3\bigg (\delta +d+\frac{\zeta _4^2}{2}\bigg )=\Lambda \end{aligned}$$

Namely

29 $$\begin{aligned} c_1&=\frac{\Lambda }{\bigg (\rho +d+\frac{\zeta _1^2}{2}\bigg )},\\ c_2&=\frac{\Lambda }{\bigg (\alpha +d+\frac{\zeta _3^2}{2}\bigg )},\\ c_3&=\frac{\Lambda }{\bigg (\delta +d+\frac{\zeta _4^2}{2}\bigg )}. \end{aligned}$$

Consequently

$$\begin{aligned} {\mathcal {L}}V_1&\le -4\left[ \left( \frac{\Lambda ^4 d\eta \alpha }{\bigg (\rho +d+\frac{\zeta _1^2}{2}\bigg )\bigg (\alpha +d+\frac{\zeta _2^3}{2}\bigg )\bigg (\delta +d+\frac{\zeta _4^2}{2}\bigg )}\right) ^{\frac{1}{4}}-\pi \right] +c_1\frac{\eta A}{N}-c_2\frac{(1-\tau )\eta VA}{NU}, \\ {\mathcal {L}}V_1&\le -4\Lambda \left[ (C^H_0 )^{1/4} -1 \right] +\frac{c_1\eta A}{N}. \end{aligned}$$

In addition, we obtain

$$\begin{aligned} V_2&=c_4(H+V+U+A+C-c_1\ln H-c_2\ln U-c_3\ln A)\\&\quad -\ln H-\ln V-\ln C+ H(t)+V(t)+U(t)+A(t)+C(t)\\&=(c_4 + 1)(H+V+U+A+C)-(c_1c_4+1) \ln H-c_2c_4 \ln U-c_3c_4 \ln A -\ln V-\ln C, \end{aligned}$$

here $c_4 > 0$ is a constant that will be decided afterward. It’s useful to illustrate that.

30 $$\begin{aligned} \liminf _{(H,V,U,A,C) \in {\mathbb {C}}_{+}^{5} \backslash U_{k}} V_{2}(H,V,U,A,C)=+\infty ,\quad \text {as}\quad k \rightarrow \infty , \end{aligned}$$

here $U_k = (\frac{1}{k}, k) \times ( \frac{1}{k}, k) \times (\frac{1}{k}, k)$. The upcoming step is to show that $V_2(H,V,U,A,C)$ has one and only one minimum value $V_2(H_0,V_0,U_0,A_0,C_0).$ $\square$

The partial derivative of $$V_2(H,V,U,A,C) $$ with respect to *H*, *V*, *U*, *A*, *C* is as follow

$$\begin{aligned} \frac{\partial V_2(H,V,U,A,C)}{\partial H}&=1+c_4-\frac{1+c_1c_4}{H},\\ \frac{\partial V_2(H,V,U,A,C)}{\partial V}&=1+c_4-\frac{1}{V},\\ \frac{\partial V_2(H,V,U,A,C)}{\partial U}&=1+c_4-\frac{c_2c_4}{U},\\ \frac{\partial V_2(H,V,U,A,C)}{\partial A}&=1+c_4-\frac{c_3c_4}{A},\\ \frac{\partial V_2(H,V,U,A,C)}{\partial C}&=1+c_4-\frac{1}{C}. \end{aligned}$$

It’s not difficult to establish that $V_2$ has a unique stagnation point.

31 $$\begin{aligned} (H(0),V(0),U(0),A(0),C(0))=\bigg (\frac{1+c_1c_4}{1+c_4},\frac{1}{1+c_4},\frac{c_2c_4}{1+c_4},\frac{c_3c_4}{1+c_4},\frac{1}{1+c_4}\bigg ). \end{aligned}$$

Moreover, the Hessian matrix of $V_2(H,V,U,A,C)$ at (*H*(0), *V*(0), *U*(0), *A*(0), *C*(0)) is

32 $$\begin{aligned} W=\begin{bmatrix} \frac{1+c_1 c_4}{H^2(0)} &{} 0 &{} 0 &{} 0 &{} 0 \\ 0 &{} \frac{1}{V^2(0)} &{} 0 &{} 0 &{} 0 \\ 0 &{} 0 &{} \frac{c_2c_4}{U^2(0)} &{} 0 &{} 0\\ 0 &{} 0 &{} 0 &{}\frac{c_3c_4}{A^2(0)} &{} 0 \\ 0 &{} 0 &{} 0 &{} 0 &{} \frac{1}{C^2(0)} \\ \end{bmatrix}. \end{aligned}$$

The Hessian matrix is evidently positive definite. As an outcome, $V_2(H,V,U,A,C)$ has a least value of *V*2(*H*, *V*, *U*, *A*, *C*)

$V_2(H(0),V(0),U(0),A(0),C(0))$. As per Eq. (30) and according to the continuity of $V_2(H,V,U,A,C)$, we can say that $V_2(H,V,U,A,C)$ has just one least value $V_2(H(0),V(0),U(0),A(0),C(0))$ contained in ${\mathbb {C}}^5_+$.

Following that, we’ll define a non-negative $C^2-$function $V : {\mathbb {C}}^5_+ \rightarrow {\mathbb {C}}_{+}$ as follows

$$\begin{aligned} V(H,V,U,A,C)=V_2(H,V,U,A,C)-V_2(H(0),V(0),U(0),A(0),C(0)). \end{aligned}$$

Considering $Ito's$ calculation and the proposed system, we reach at

33 $$ \begin{aligned} {\mathcal {L}}(V)&\le c_4 \bigg \{-4\Lambda \bigg [({\tilde{C}}^H_0 )^{1/4}-1\bigg ] +\frac{c_1\eta A}{N}\bigg \} -\frac{\Lambda }{H}+\frac{\eta A}{N}+(\rho +d)\\&\quad +\frac{\zeta _1^2}{2}-\frac{\rho H}{V}+\frac{(1-\tau )\eta A}{N}+d+\frac{\zeta _2^2}{2}\\&\quad -\frac{\delta A}{C}+d+\frac{\zeta _5^2}{2}+\Lambda -d(H(t)+V(t)+U(t)+A(t)+C(t)), \end{aligned}$$

as a consequence of which the preceding assumption can be formed:

34 $$\begin{aligned} {\mathcal {L}}V&\le -c_4c_5+(c_1c_4+1)\frac{\eta A}{N}-\frac{\Lambda }{H}+(\rho +d)\\&\quad +\frac{\zeta _1^2}{2}-\frac{\rho H}{V}+\frac{(1-\tau )\eta A}{N}+d+\frac{\zeta _2^2}{2}-\frac{\delta A}{C}+d+\frac{\zeta _5^2}{2}+\Lambda ,\\&\quad -d(H(t)+V(t)+U(t)+A(t)+C(t)) \end{aligned}$$

where

$$\begin{aligned} C_5 = 4\Lambda \bigg [(C^H_0 )^{1/4} -1\bigg ] > 0. \end{aligned}$$

The subsequent step is to establish the set

$$\begin{aligned} D =\bigg \{\epsilon _1< H< \frac{1}{\epsilon _2},~~\epsilon _1< V<\frac{1}{\epsilon _2},~~ \epsilon _1< U<\frac{1}{\epsilon _2},~~\epsilon _1< A<\frac{1}{\epsilon _2}~~\epsilon _1< C <\frac{1}{\epsilon _2}\bigg \}, \end{aligned}$$

where $\epsilon _i>0$ is a negligibly minor constant to be found afterwards for $(i = 1,2)$. We’ll split the entire ${\mathbb {C}}_{+}^{5} \backslash D$ into the preceding regions for clarity’s reason.

$$ \begin{aligned} D_1&=\bigg \{(H,V,U,A,C)\in {\mathbb {C}}_{+}^{5}, 0< H \le \epsilon _1\bigg \},\\ D_2&= \bigg \{(H,V,U,A,C) \in {\mathbb {C}}_{+}^{5}, 0< V \le \epsilon _2, H> \epsilon _2\bigg \}, \\ D_3&=\bigg \{(H,V,U,A,C) \in {\mathbb {C}}_{+}^{5}, 0<U \le \epsilon _1, V>\epsilon _2\bigg \},\\ D_4&= \bigg \{(H,V,U,A,C)\in {\mathbb {C}}_{+}^{5}, 0<A \le \epsilon _1, U> \epsilon _2\bigg \}, \\ D_5&= \bigg \{(H,V,U,A,C)\in {\mathbb {C}}_{+}^{5}, 0<C \le \epsilon _1, A > \epsilon _2\bigg \}, \\ D_6&=\bigg \{(H,V,U,A,C) \in {\mathbb {C}}_{+}^{5}, H \ge \frac{1}{\epsilon _2}\bigg \},\\ D_7&=\bigg \{(H,V,U,A,C)\in {\mathbb {C}}_{+}^{5}, A \ge \frac{1}{\epsilon _2}\bigg \},\\ D_8&=\bigg \{(H,V,U,A,C) \in {\mathbb {C}}_{+}^{5}, C \ge \frac{1}{\epsilon _2}\bigg \},\\ D_9&=\bigg \{(H,V,U,A,C)\in {\mathbb {C}}_{+}^{5}, V \ge \frac{1}{\epsilon _2}\bigg \},\\ D_{10}&=\bigg \{(H,V,U,A,C)\in {\mathbb {C}}_{+}^{5}, V \ge \frac{1}{\epsilon _2}\bigg \}. \end{aligned}$$

Now we’ll show that $$LV(H,V,U,A,C) < 0 $$ on $${\mathbb {C}}_{+}^{5} \backslash D$$, which is the similar as presenting it on the ten regions specified earlier.

**Case 1.** If $(H,V,U,A,C) \in D_1$, so by Eq. (34), it gives

$$\begin{aligned} {\mathcal {L}}V&\le -c_4c_5+(c_1c_4+1)\frac{\eta A}{N}-\frac{\Lambda }{H}+(\rho +d)+\frac{\zeta _1^2}{2}-\frac{\rho H}{V}\\&\quad +\frac{(1-\tau )\eta A}{N}+d+\frac{\zeta _2^2}{2}-\frac{\delta A}{C}+d+\frac{\zeta _5^2}{2}+\Lambda -d N,\\&\le (c_1c_4+1)\frac{\eta A}{N}-\frac{\Lambda }{\epsilon _1}+(\rho +d)+\frac{\zeta _1^2}{2}-\frac{\rho H}{V}+\frac{(1-\tau )\eta A}{N}+d+\frac{\zeta _2^2}{2}-\frac{\delta A}{C}+d+\frac{\zeta _5^2}{2}+\Lambda .\\ \end{aligned}$$

Choosing $\epsilon _1 > 0,$ yields ${\mathcal {L}}V<0$ for each $(H,V,U,A,C)\in D_1.$

**Case 2.** If $(H,V,U,A,C)\in D_2$, then from Eq. (34), we can obtain

$$\begin{aligned} {\mathcal {L}}V&\le -c_4c_5+(c_1c_4+1)\frac{\eta A}{N}-\frac{\Lambda }{H}+(\rho +d)+\frac{\zeta _1^2}{2}-\frac{\rho H}{V}\\&\quad +\frac{(1-\tau )\eta A}{N}+d+\frac{\zeta _2^2}{2}-\frac{\delta A}{C}+d+\frac{\zeta _5^2}{2}+\Lambda -d N,\\&\le -c_4c_5+(c_1c_4+1)\frac{\eta A}{N}+(\rho +d)+\frac{\zeta _1^2}{2}-\frac{\rho H}{V}+\frac{(1-\tau )\eta A}{N}+d+\frac{\zeta _2^2}{2}-\frac{\delta A}{C}+d+\frac{\zeta _5^2}{2}+\Lambda -d \epsilon _1.\\ \end{aligned}$$

Let $\epsilon _1 > 0$, so we can get ${\mathcal {L}}V<0$ for any $(H,V,U,A,C)\in D_2.$

**Case 3.** If $(H,V,U,A,C)\in D_3$, then from Eq. (34), we obtain

$$\begin{aligned}  {\mathcal {L}}V&\le -c_4c_5+(c_1c_4+1)\frac{\eta A}{N}-\frac{\Lambda }{H}+(\rho +d)+\frac{\zeta _1^2}{2}-\frac{\rho H}{V}\\&\quad +\frac{(1-\tau )\eta A}{N}+d+\frac{\zeta _2^2}{2}-\frac{\delta A}{C}+d+\frac{\zeta _5^2}{2}+\Lambda -d N,\\&\le (c_1c_4+1)\frac{\eta A}{N}+(\rho +d)+\frac{\zeta _1^2}{2}-\frac{\rho H}{V}+\frac{(1-\tau )\eta A}{N}+d+\frac{\zeta _2^2}{2}-\frac{\delta A}{C}+d+\frac{\zeta _5^2}{2}+\Lambda -d \frac{\epsilon _2}{\epsilon _1}.\\ \end{aligned}$$

By taking small $\epsilon _1,\epsilon _2 > 0,$ so, as ${\mathcal {L}}V<0$ for each $(H,V,U,A,C)\in D_3.$

**Case 4.** $If (H,V,U,A,C)\in D_4,$ from Eq. (34), we obtain

$$ \begin{aligned} {\mathcal {L}}V&\le -c_4c_5+(c_1c_4+1)\frac{\eta A}{N}-\frac{\Lambda }{H}+(\rho +d)+\frac{\zeta _1^2}{2}-\frac{\rho H}{V}\\&\quad +\frac{(1-\tau )\eta A}{N}+d+\frac{\zeta _2^2}{2}-\frac{\delta A}{C}+d+\frac{\zeta _5^2}{2}+\Lambda -d N,\\&\le (c_1c_4+1)\frac{\eta A}{N}-\frac{\Lambda }{\epsilon _1}+(\rho +d)+\frac{\zeta _1^2}{2}-\frac{\rho H}{V}+\frac{(1-\tau )\eta A}{N}+d+\frac{\zeta _2^2}{2}-\frac{\delta A}{C}+d+\frac{\zeta _5^2}{2}+\Lambda -d\epsilon _1.\\ \end{aligned}$$

If we pick a small enough $\epsilon _1 > 0$, then we get ${\mathcal {L}}V<0$ for every $(H,V,U,A,C)\in D_4.$.

**Case 5.** If $(H,V,U,A,C) \in D_5,$ from Eq. (34), we obtain

$$\begin{aligned}  {\mathcal {L}}V&\le -c_4c_5+(c_1c_4+1)\frac{\eta A}{N}-\frac{\Lambda }{H}+(\rho +d)+\frac{\zeta _1^2}{2}-\frac{\rho H}{V}\\&\quad +\frac{(1-\tau )\eta A}{N}+d+\frac{\zeta _2^2}{2}-\frac{\delta A}{C}+d+\frac{\zeta _5^2}{2}+\Lambda -d N,\\&\le (c_1c_4+1)\frac{\eta A}{N}-\frac{\Lambda }{\epsilon _1}+(\rho +d)+\frac{\zeta _1^2}{2}+\frac{(1-\tau )\eta A}{N}+d+\frac{\zeta _2^2}{2}-\frac{\delta A}{\epsilon _2}+d+\frac{\zeta _5^2}{2}+\Lambda .\\ \end{aligned}$$

We take quite small $\epsilon _2 > 0$, so now we can get ${\mathcal {L}}V<0$ for any $(H,V,U,A,C)\in D_5.$

**Case 6.** If $(H,V,U,A,C)\in D_6,$ from Eq. (34), we obtain

$$\begin{aligned}  {\mathcal {L}}V&\le -c_4c_5+(c_1c_4+1)\frac{\eta A}{N}-\frac{\Lambda }{H}+(\rho +d)+\frac{\zeta _1^2}{2}-\frac{\rho H}{V}\\&\quad +\frac{(1-\tau )\eta A}{N}+d+\frac{\zeta _2^2}{2}-\frac{\delta A}{C}+d+\frac{\zeta _5^2}{2}+\Lambda -d N,\\&\le (c_1c_4+1)\frac{\eta A}{N}-\frac{\Lambda }{\epsilon _1}+(\rho +d)+\frac{\zeta _1^2}{2}+\frac{(1-\tau )\eta A}{N}+d+\frac{\zeta _2^2}{2}-\frac{\delta A}{C}+d+\frac{\zeta _5^2}{2}+\Lambda -\frac{d}{\epsilon _2}.\\ \end{aligned}$$

We can choose sufficiently small $\epsilon _2, \epsilon _1 > 0$, so now we can get ${\mathcal {L}}V<0$ for any $(H,V,U,A,C)\in D_6.$

**Case 7.** If $(H,V,U,A,C)\in D_7,$ from Eq. (34), we obtain

$$\begin{aligned}  {\mathcal {L}}V&\le -c_4c_5+(c_1c_4+1)\frac{\eta A}{N}-\frac{\Lambda }{H}+(\rho +d)+\frac{\zeta _1^2}{2}-\frac{\rho H}{V}\\&\quad +\frac{(1-\tau )\eta A}{N}+d+\frac{\zeta _2^2}{2}-\frac{\delta A}{C}+d+\frac{\zeta _5^2}{2}+\Lambda -\frac{d}{\epsilon _2},\\&\le (c_1c_4+1)\frac{\eta A}{N}-\frac{\Lambda }{\epsilon _1}+(\rho +d)+\frac{\zeta _1^2}{2}+\frac{(1-\tau )\eta A}{N}+d+\frac{\zeta _2^2}{2}-\frac{\delta A}{C}+d+\frac{\zeta _5^2}{2}+\Lambda -\frac{d}{\epsilon _2}.\\ \end{aligned}$$

By selecting smallest value of $\epsilon _2 > 0$, so we can get ${\mathcal {L}}V<0$ for any $(H,V,U,A,C)\in D_7.$

**Case 8.** If $(H,V,U,A,C)\in D_8,$ from Eq. (34), we obtain

$$\begin{aligned}  {\mathcal {L}}V&\le -c_4c_5+(c_1c_4+1)\frac{\eta A}{N}-\frac{\Lambda }{H}+(\rho +d)+\frac{\zeta _1^2}{2}-\frac{\rho H}{V}\\&\quad +\frac{(1-\tau )\eta A}{N}+d+\frac{\zeta _2^2}{2}-\frac{\delta A}{C}+d+\frac{\zeta _5^2}{2}+\Lambda -d N,\\&\le (c_1c_4+1)\frac{\eta A}{N}-\frac{\Lambda }{\epsilon _1}+(\rho +d)+\frac{\zeta _1^2}{2}+\frac{(1-\tau )\eta A}{N}+d+\frac{\zeta _2^2}{2}-\frac{\delta A}{C}+d+\frac{\zeta _5^2}{2}+\Lambda -\frac{d}{\epsilon _2}.\\ \end{aligned}$$

Let we take the smallest value of $\epsilon _2 > 0$, so we can get ${\mathcal {L}}V<0$ for any $(H,V,U,A,C)\in D_8.$

**Case 9.** If $(H,V,U,A,C)\in D_9,$ from Eq. (34), we obtain

$$\begin{aligned} {\mathcal {L}}V&\le -c_4c_5+(c_1c_4+1)\frac{\eta A}{N}-\frac{\Lambda }{H}+(\rho +d)+\frac{\zeta _1^2}{2}-\frac{\rho H}{V}+\frac{(1-\tau )\eta A}{N}\\&\quad +d+\frac{\zeta _2^2}{2}-\frac{\delta A}{C}+d+\frac{\zeta _5^2}{2}+\Lambda -d N,\\&\le -c_4c_5+(c_1c_4+1)\frac{\eta A}{N}-\frac{\Lambda }{\epsilon _1}+(\rho +d) +\frac{\zeta _1^2}{2}-\rho \frac{\epsilon _2}{\epsilon _1}+\frac{(1-\tau )\eta A}{N}\\&\quad +d+\frac{\zeta _2^2}{2}-\frac{\delta A}{C}+d+\frac{\zeta _5^2}{2}+\Lambda -d N.\\ \end{aligned}$$

Now if $\epsilon _1 > 0$, so now we can find ${\mathcal {L}}V<0$ for every $(H,V,U,A,C)\in D_9.$

**Case 10.** If $(H,V,U,A,C)\in D_10,$ from Eq. (34), we obtain

$$\begin{aligned} {\mathcal {L}}V&\le -c_4c_5+(c_1c_4+1)\frac{\eta A}{N}-\frac{\Lambda }{H}+(\rho +d)+\frac{\zeta _1^2}{2}-\frac{\rho H}{V}+\frac{(1-\tau )\eta A}{N}\\&\quad +d+\frac{\zeta _2^2}{2}-\frac{\delta A}{C}+d+\frac{\zeta _5^2}{2}+\Lambda -d N,\\&\le -c_4c_5+(c_1c_4+1)\frac{\eta A}{N}-\frac{\Lambda }{\epsilon _1}+(\rho +d)+\frac{\zeta _1^2}{2}-\frac{\rho H}{V} +\frac{(1-\tau )\eta A}{N}\\&\quad +d+\frac{\zeta _2^2}{2}-\frac{\delta A}{C}+d+\frac{\zeta _5^2}{2}+\Lambda -\frac{d}{\epsilon _2}.\\ \end{aligned}$$

For the smallest velue of $\epsilon _2 > 0$, so we can obtain ${\mathcal {L}}V<0$ for any arbitrary $(H,V,U,A,C)\in D_10.$

As an outcome, we establish that a constant $W > 0$ is such that which assures

$$\begin{aligned} LV(H,V,U,A,C)< -W < 0~\hbox {for all}~(H,V,U,A,C) \in {\mathbb {C}}_{+}^{5} \backslash D. \end{aligned}$$

Hence

35 $$\begin{aligned} dV(H,V,U,A,C)&< -Wdt + [(c_4 + 1)H-(c_1c_4 + 1)\zeta 1]dW_1(t) + [(c_4+ 1)V-\zeta _2]dW_2(t) \\&\quad + [(c_4+ 1)U-c_2c_4\zeta _3]dW_3(t)+ [(c_4 + 1)A -c_3c_4\zeta _4]dW_4(t)\\&\quad + [(c_4 + 1)C-\zeta _5]dW_5(t). \end{aligned}$$

Assume that $(H(0),V(0), U(0), A(0), C(0)) = (x_1, x_2, x_3,x_4,x_5) = x \in {\mathbb {C}}_{+}^{5} \backslash D$, and $\tau ^x$ is that time for which a path start from *x* reach to the set *D*,

$$\begin{aligned} \tau _n= inf\{t : |X(t)| = n\}~\hbox {and}~\tau ^{(n)}(t) = \min \{\tau ^x,t,\tau _n\}. \end{aligned}$$

One can get the following by taking integration of the both hand sides of the inequality (35) from zero to $\tau ^{(n)}(t)$, considering expectation, and utilizing Dynkin’s calculation.

$$\begin{aligned}&{\mathbb {U}}V(H(\tau ^{(n)}(t)), V(\tau ^{(n)}(t)), U(\tau ^{(n)}(t)), A(\tau ^{(n)}(t)), C(\tau ^{(n)}(t)))-V(x)\\&\quad = {\mathbb {U}}\int ^{\tau (n)(t)}_0 LV(H(u), V(u), U(u), A(u), C(u))du,\\&\quad \le {\mathbb {U}}\int ^{\tau (n)(t)}_0 -W du = -W{\mathbb {U}}\tau ^{(n)}(t). \end{aligned}$$

As *V*(*x*) is a non-negative, therefore

$$\begin{aligned} {\mathbb {U}}\tau ^{(n)}(t) \le \frac{V(x)}{W}. \end{aligned}$$

We have $P\{\tau _e = \infty \} = 1$ as a result of the proof of Theorem 3. Conversely, the system (1) can be defined as regular. As a result, if we take $t \rightarrow \infty$ and $n \rightarrow \infty$, we almost certainly obtain $\tau {(n)}(t) \rightarrow \tau ^x$ almost certainly.

As a result, using Fatou’s lemma, we arrive at

$$\begin{aligned} {\mathbb {U}}\tau ^{(n)}(t) \le \frac{V(x)}{W}< \infty \end{aligned}$$

Clearly, $sup_{x\in K} {\mathbb {U}}\tau ^x < \infty ,$ here *K* is a compact subset from ${\mathbb {C}}_{+}^{5}$. It confirms Lemma 5 condition 2.

Additionally, the diffusion matrix of the system (1) is

$$\begin{aligned} W=\begin{bmatrix} \zeta ^2_1H^2 &{} 0 &{} 0 &{} 0 &{} 0\\ 0 &{} \zeta ^2_2 V^2 &{} 0 &{} 0 &{} 0\\ 0 &{} 0 &{} \zeta ^2_3 U^2 &{} 0 &{} 0\\ 0 &{} 0 &{} 0 &{} \zeta ^2_4 A^2 &{} 0 \\ 0 &{} 0 &{} 0 &{} 0 &{} \zeta ^2_5 C^2 \\ \end{bmatrix} \end{aligned}$$

Choosing $M = \min _{(H,V,U,A,C)\in {\overline{D}}\in C^5_+}\{\zeta ^2_1H^2,\zeta ^2_2 V^2,\zeta ^2_3U^2, \zeta ^2_4A^2, \zeta ^2_5C^2\},$ we obtain

$$\begin{aligned} \sum ^5_{i,j=1} a_{ij}(H,V,U,A,C)\rho _i\rho _j= & {} \zeta ^2_1H^2 \rho ^2+\zeta ^2_2 V^2\rho _2^2+\zeta ^2_3U^2\rho ^2\\&+\zeta ^2_4\rho _4^2A^2+\zeta ^2_5\rho _5^2C^2\ge M|\rho |^2,(H,V,U,A,C) \in {\overline{D}}, \end{aligned}$$

where $\rho = (\rho _1, \rho _2, \rho _3,\rho _4,\rho _5) \in {\mathbb {C}}_{+}^{5}.$ This implies that Lemma 5 condition 1 is likewise true. Following to the preceding analysis, Lemma 5 indicates that the system (1) is ergodic and has just one stationary distribution.

## A fractal–fractional NoV model with Mittag–Leffler kernel

Fractional calculus (FC) has gained much interest from the researcher and scientists, because of its uses in different fields of real-world problems than that of integer order. FC has taken advantages and popularity of modelling with memory effects^30,31^. Inspired from the work of Atangana^25,31–33^, in the field of FC, we convert the proposed norovirus model to investigate its parameter with the available data in system (1). The current section, deals with the approach of Atangana–Baleanu fractal–fractional (FF) derivative operator having fractional order ${p}$ and fractal dimension ${q}$. We consider the presented model (2) to fractal–fractional order in sense of $\mathcal {ABC}$ operator. It is due to the fractional order derivatives have extra degree of freedom and some other characteristics of heredity, memory, and description of the past as well as present and future. This operator has non-singular kernel and is non-local operator. The NoV model can be shown through the following fractal–fractional differential system:

36 $$\begin{aligned} ^{\mathcal {ABC}}{\mathbf {D}}^{{{p},{q}}}H&=\Lambda -\frac{\eta {H}(t)A(t)}{N}-(\rho +d) H(t),\\ ^{\mathcal {ABC}}{\mathbf {D}}^{{{p},{q}}}V&=\rho {H}(t)-\frac{(1-\tau )\eta {V}(t)A(t)}{N}-d V(t),\\ ^{\mathcal {ABC}}{\mathbf {D}}^{{{p},{q}}}U&=\frac{\eta {H}(t)A(t)}{N}+\frac{(1-\tau )\eta V(t)A(t)}{N}-(\alpha +d)U(t),\\ ^{\mathcal {ABC}}{\mathbf {D}}^{{{p},{q}}}A&=\alpha U(t)-(\delta +d) A(t),\\ ^{\mathcal {ABC}}{\mathbf {D}}^{{{p},{q}}}C&=\delta A(t)-d C(t). \end{aligned}$$

Further, the system (36) can be write in the ABC fractal–fractional into the following way

37 $$\begin{aligned} \left\{ \begin{aligned}&^{\mathcal {ABC}}{\mathbf {D}}^{p}(H(t))={q}t^{{q}-1}\mathcal {K}_1\big (H(t),t\big )=\Lambda -\frac{\eta {H}(t)A(t)}{N}-(\rho +d) H(t), \\&^{\mathcal {ABC}}{\mathbf {D}}^{p}({V}(t))={q}t^{{q}-1}\mathcal {K}_2\big ({V}(t),t\big )=\rho {H}(t)-\frac{(1-\tau )\eta {V}(t)A(t)}{N}-d V(t), \\&^{\mathcal {ABC}}{\mathbf {D}}^{p}({U}(t))={q}t^{{q}-1}\mathcal {K}_3\big ({U}(t),t\big )=\frac{\eta {H}(t)A(t)}{N}+\frac{(1-\tau )\eta V(t)A(t)}{N}-(\alpha +d)U(t),\\&^{\mathcal {ABC}}{\mathbf {D}}^{p}({A}(t))={q}t^{{q}-1}\mathcal {K}_4\big ({A}(t),t\big )=\alpha U(t)-(\delta +d) A(t),\\&^{\mathcal {ABC}}{\mathbf {D}}^{p}({C}(t))={q}t^{{q}-1}\mathcal {K}_5\big ({C}(t),t\big )=\delta A(t)-d C(t), \end{aligned}\right. \end{aligned}$$

where $\mathcal {K}_i, i=1,2,3,4,5$. In view of Eq. (37)

38 $$\begin{aligned} ^{\mathcal {ABC}}{\mathbf {D}}^{{p}}\Upsilon (t)= & {} {q}t^{{q}-1}\Psi \big (t,\Upsilon (t)\big )\nonumber \\ \Upsilon (0)= & {} \Upsilon _0,\ \ t\in \Upsilon \end{aligned}$$

along with

$$\begin{aligned} \Upsilon (t)-\Upsilon _0=\frac{1-{p}}{\mathcal {ABC}({p})}t^{{q}-1}\big [\Psi \big (t,\Upsilon (t)\big )\big ]+\frac{{q}{p}}{\mathcal {ABC}({p})\Gamma ({p})}\int _0^{t} ({t}-\wp )^{{p}-1}\wp ^{{q}-1}\Psi \big (\Upsilon (\wp ),\wp \big )d\wp . \end{aligned}$$

where

39 $$\begin{aligned} \left\{ \begin{aligned}&\Upsilon :=(H,V,U,A,C)^T,\\&\Upsilon _0:=(H_0,V_0,U_0,A_0,C_0)^T,\\&\Upsilon (t,\mho (t)):=(\mathcal {K}_i(t,H,V,U,A,C))^T, \ \ i=1,2,3,4,5. \end{aligned}\right. \end{aligned}$$

Taking the first equation of (37) and by using the anti-derivative of fractal dimension and fractional order in sense of, we have

$$\begin{aligned} H(t)-H_0=\frac{1-{p}}{\mathcal {ABC}({p})}t^{{q}-1}\big [\mathcal {K}_1\big (H(t),t\big )\big ]+\frac{{q}{p}}{\mathcal {ABC}({p})\Gamma ({p})}\int _0^{t} ({t}-\wp )^{{p}-1}\wp ^{{q}-1}\mathcal {K}_1\big (H(\wp ),\wp \big )d\wp . \end{aligned}$$

by letting $t=t_{\flat +1}$ for $\flat =0,1,2 \ldots ,$

$$\begin{aligned} H(t_{\flat +1})-H_0= & {} \frac{(1-{p})}{\mathcal {ABC}({p})}(t_{\flat +1}^{{q}-1}) \bigg [\mathcal {K}_1\big (H(t_{\flat }),t_{\flat }\big )\bigg ]\\&+\frac{{q}{p}}{\mathcal {ABC}({p})\Gamma ({p})}\int _0^{t_{\flat +1}} ({t_{\flat +1}}-\wp )^{{p}-1}\wp ^{{q}-1}\mathcal {K}_1\big (H(\wp ),(\wp )\big )d\wp ,\\= & {} \frac{(1-{p})}{\mathcal {ABC}({p})}(t_{\flat +1}^{{q}-1})\bigg [\mathcal {K}_1\big (H(t_{\flat }), t_{\flat }\big )\bigg ]\\&+\frac{{q}{p}}{\mathcal {ABC}({p})\Gamma ({p})}\sum _{\varsigma =0}^\flat \int _\varsigma ^{t_{\varsigma +1}}({t_{\flat +1}}-\wp )^{{p}-1}\wp ^{{q}-1}\mathcal {K}_1\big (H(\wp ),\wp \big )d\wp . \end{aligned}$$

The approximate function be $\mathcal {K}_1$ on the interval $[{t}_\varsigma ,{t}_{\varsigma +1}]$ through the interpolation polynomial as follows

$$\begin{aligned} \mathcal {K}_1\cong \frac{\mathcal {K}_1}{\Delta }({t}-{t}_{\varsigma -1})- \frac{{\mathfrak {R}}_1}{\Delta }({t}-{t}_{\varsigma }) \end{aligned}$$

which implies that

40 $$\begin{aligned} H(t_{\flat +1})= & {} H_0+\frac{(1-{p})}{\mathcal {ABC}({p})}(t_{\flat +1}^{{q}-1})\bigg [\mathcal {K}_1\big (H(t_{\flat }),t_{\flat }\big )\bigg ]+\frac{{q}{p}}{\mathcal {ABC}({p})\Gamma ({p})}\sum _{\varsigma =0}^\flat \bigg (\frac{\mathcal {K}_1\big (H(t_{\flat }),t_{\flat }\big )}{\Delta }\nonumber \\&\times \int _\varsigma ^{t_{\varsigma +1}}(t-t_{\varsigma -1})({t_{\varsigma +1}}-t)^{{p}-1}t_\varsigma ^{{q}-1}dt-\frac{\mathcal {K}_1\big (H(t_{\flat }),t_{\flat }\big )}{\Delta }\int _\varsigma ^{t_{\varsigma +1}}(t-t_{\varsigma })({t_{\flat +1}}-t)^{{p}-1}t_\varsigma ^{{q}-1}dt\bigg ), \nonumber \\ H(t_{\flat +1})= & {} H_0+\frac{(1-{p})}{\mathcal {ABC}({p})}(t_{\flat +1}^{{q}-1})\bigg [\mathcal {K}_1\big (H(t_{\flat }),t_{\flat }\big )\bigg ]+\frac{{q}{p}}{\mathcal {ABC}({p})\Gamma ({p})}\sum _{\varsigma =0}^\flat \bigg (\frac{t_\varsigma ^{{q}-1}\mathcal {K}_1\big (H(t_{\varsigma }),t_{\varsigma }\big )}{\Delta }{\mathbf {I}}_{\varsigma -1, {p}}\nonumber \\&-\frac{t_{\varsigma -1}^{{q}-1}\mathcal {K}_1\big (H(t_{\varsigma -1}),t_{\varsigma -1}\big )}{\Delta }{\mathbf {I}}_{\varsigma , {p}}\bigg ). \end{aligned}$$

Calculating ${\mathbf {I}}_{\varsigma -1, {p}}$ and ${\mathbf {I}}_{\varsigma , {p}}$ we get

$$\begin{aligned} {\mathbf {I}}_{\varsigma -1,{p}}= & {} \int _\varsigma ^{t_{\varsigma +1}}(t-t_{\varsigma -1}) (t_{\flat +1}-t)^{{p}-1}dt,\\= & {} -\frac{1}{{p}}\bigg [(t_{\varsigma +1}-t_{\varsigma -1})(t_{\flat +1}-t_{\varsigma +1})^{p}-(t_\varsigma -t_{\varsigma -1})(t_{\flat +1}-t_\varsigma )^{p}\bigg ]\\&-\frac{1}{{p}({p}-1)}\bigg [(t_{\flat +1}-t_{\varsigma +1})^{{p}+1}-(t_{\flat +1}-t_\varsigma )^{{p}+1}\bigg ], \end{aligned}$$

and

$$\begin{aligned} {\mathbf {I}}_{\varsigma ,{p}}= & {} \int _\varsigma ^{t_{\varsigma +1}}(t-t_{\varsigma })(t_{\flat +1}-t)^{{p}-1}dt,\\= & {} -\frac{1}{{p}}\bigg [(t_{\varsigma +1}-t_{\varsigma })(t_{\flat +1}-t_{\varsigma +1})^{p}\bigg ]\\&-\frac{1}{{p}({p}-1)}\bigg [(t_{\flat +1}-t_{\varsigma +1})^{{p}+1}-(t_{\flat 
+1}-t_\varsigma )^{{p}+1}\bigg ], \end{aligned}$$

put $t_\varsigma =\varsigma \Delta ,$ we get

41 $$\begin{aligned} {\mathbf {I}}_{\varsigma -1,{p}}= & {} -\frac{\Delta ^{{p}+1}}{{p}}\bigg [(\varsigma +1-(\varsigma -1))(\flat +1-(\varsigma +1))^{p}-(\varsigma -(\varsigma -1))(\flat +1-\varsigma ^{p})\bigg ]\nonumber \\&-\frac{\Delta ^{{p}+1}}{{p}({p}-1)}\bigg [(\flat +1-(\varsigma +1))^{{p}+1}-(\flat +1-\varsigma )^{{p}+1}\bigg ],\nonumber \\= & {} \frac{\Delta ^{{p}+1}}{{p}({p}-1)}\bigg [-2({p}+1)(\flat -\varsigma )^{p}+({p}+1)(\flat +1-\varsigma )^{p}-(\flat -\varsigma )^{{p}+1}+(\flat +1-\varsigma )^{{p}+1}\bigg ],\nonumber \\= & {} \frac{\Delta ^{{p}+1}}{{p}({p}-1)}\bigg [(\flat -\varsigma )^{p}(-2({p}+1)-(\flat -\varsigma ))+(\flat +1-\varsigma )^{p}({p}+1+\flat +1-\varsigma )\bigg ],\nonumber \\= & {} \frac{\Delta ^{{p}+1}}{{p}({p}-1)}\bigg [(\flat +1-\varsigma )^{p}(\flat -\varsigma +2+{p})-(\flat -\varsigma )^{p}(\flat -\varsigma +2+2{p})\bigg ], \end{aligned}$$

and

42 $$\begin{aligned} {\mathbf {I}}_{\varsigma ,{p}}= & {} -\frac{\Delta ^{{p}+1}}{{p}}\bigg [(\varsigma +1-\varsigma )(\flat +1-(\varsigma +1))^{p}\bigg ]-\frac{\Delta ^{{p}+1}}{{p}({p}-1)}\bigg [(\flat +1-(\varsigma +1))^{{p}+1}-(\flat +1-\varsigma )^{{p}+1}\bigg ],\nonumber \\= & {} \frac{\Delta ^{{p}+1}}{{p}({p}-1)}\bigg [-({p}+1)(\flat -\varsigma )^{p}-(\flat -\varsigma )^{{p}+1}+(\flat +1-\varsigma )^{{p}+1}\bigg ],\nonumber \\= & {} \frac{\Delta ^{{p}+1}}{{p}({p}-1)}\bigg [(\flat -\varsigma )^{p}(-(\varsigma +1)-(\flat -\varsigma ))+(\flat +1-\varsigma )^{{p}+1}\bigg ],\nonumber \\= & {} \frac{\Delta ^{{p}+1}}{{p}({p}-1)}\bigg [(\flat +1-\varsigma )^{{p}+1}-(\flat -\varsigma )^{p}(\flat -\varsigma +1+{p})\bigg ], \end{aligned}$$

substituting the values of (41) and (42) in (40), we obtain

43 $$\begin{aligned} H(t_{\flat +1})=\left\{ \begin{aligned}&H_0+\frac{(1-{p})}{\mathcal {ABC}({p})}(t_{\flat +1}^{{q}-1})\bigg [\mathcal {K}_1\big (H(t_{\flat }),t_{\flat }\big )\bigg ]+\frac{{q}{p}}{\mathcal {ABC}({p})\Gamma ({p})}\sum _{\varsigma =0}^\flat \bigg (\frac{t_\varsigma ^{{q}-1}\mathcal {K}_1\big (H(t_{\varsigma }),t_{\varsigma }\big )}{\Delta }\\&\times \bigg [\frac{\Delta ^{{p}+1}}{{p}({p}-1)}\bigg [(\flat +1-\varsigma )^{p}(\flat -\varsigma +2+{p})-(\flat -\varsigma )^{p}(\flat -\varsigma +2+2{p})\bigg ]\bigg ]\\&-\frac{t_{\varsigma -1}^{{q}-1}\mathcal {K}_1\big (H(t_{\varsigma -1}),t_{\varsigma -1}\big )}{\Delta }\bigg [\frac{\Delta ^{{p}+1}}{{p}({p}-1)}\bigg [(\flat +1-\varsigma )^{{p}+1}-(\flat -\varsigma )^{p}(\flat -\varsigma +1+{p})\bigg ]\bigg ]\bigg ). \end{aligned}\right. \end{aligned}$$

And similarly for the other classes *V*, *U*, *A* and *C* we may find the same scheme as

44 $$\begin{aligned} {V}(t_{\flat +1})= & {} \left\{ \begin{aligned}&{V}_0+\frac{(1-{p})}{\mathcal {ABC}({p})}(t_{\flat +1}^{{q}-1})\bigg [\mathcal {K}_2\big ({V}(t_{\flat }),t_{\flat }\big )\bigg ]+\frac{{q}{p}}{\mathcal {ABC}({p})\Gamma ({p})}\sum _{\varsigma =0}^\flat \bigg (\frac{t_\flat ^{{q}-1}\mathcal {K}_2\big ({V}(t_{\varsigma }),t_{\varsigma }\big )}{\Delta }\\&\times \bigg [\frac{\Delta ^{{p}+1}}{{p}({p}-1)}\bigg [(\flat +1-\varsigma )^{p}(\flat -\varsigma +2+{p})-(\flat -\varsigma )^{p}(\flat -\varsigma +2+2{p})\bigg ]\bigg ]\\&-\frac{t_{\varsigma -1}^{{q}-1}\mathcal {K}_2\big ({V}(t_{\varsigma -1}),t_{\varsigma -1}\big )}{\Delta }\bigg [\frac{\Delta ^{{p}+1}}{{p}({p}-1)}\bigg [(\flat +1-\varsigma )^{{p}+1}-(\flat -\varsigma )^{p}(\flat -\varsigma +1+{p})\bigg ]\bigg ]\bigg ). \end{aligned}\right. \end{aligned}$$

45 $$\begin{aligned} {U}(t_{\flat +1})= & {} \left\{ \begin{aligned}&{U}_0+\frac{(1-{p})}{\mathcal {ABC}({p})}(t_{\flat +1}^{{q}-1})\bigg [\mathcal {K}_3\big ({U}(t_{\flat }),t_{\flat }\big )\bigg ]+\frac{{q}{p}}{\mathcal {ABC}({p})\Gamma ({p})}\sum _{\varsigma =0}^\flat \bigg (\frac{t_\varsigma ^{{q}-1}\mathcal {K}_3\big ({U}(t_{\varsigma }),t_{\varsigma }\big )}{\Delta }\\&\times \bigg [\frac{\Delta ^{{p}+1}}{{p}({p}-1)}\bigg [(\flat +1-\varsigma )^{p}(\flat -\varsigma +2+{p})-(\flat -\varsigma )^{p}(\flat -\varsigma +2+2{p})\bigg ]\bigg ]\\&-\frac{t_{\varsigma -1}^{{q}-1}\mathcal {K}_3\big ({U}(t_{\varsigma -1}),t_{\varsigma -1}\big )}{\Delta }\bigg [\frac{\Delta ^{{p}+1}}{{p}({p}-1)}\bigg [(\flat +1-\varsigma )^{{p}+1}-(\flat -\varsigma )^{p}(\flat -\varsigma +1+{p})\bigg ]\bigg ]\bigg ). \end{aligned}\right. \end{aligned}$$

46 $$\begin{aligned} {A}(t_{\flat +1})= & {} \left\{ \begin{aligned}&{A}_0+\frac{(1-{p})}{\mathcal {ABC}({p})}(t_{\flat +1}^{{q}-1})\bigg [\mathcal {K}_4\big ({A}(t_{\flat }),t_{\flat }\big )\bigg ]+\frac{{q}{p}}{\mathcal {ABC}({p})\Gamma ({p})}\sum _{\varsigma =0}^\flat \bigg (\frac{t_\varsigma ^{{q}-1}\mathcal {K}_4\big ({A}(t_{\varsigma }),t_{\varsigma }\big )}{\Delta }\\&\times \bigg [\frac{\Delta ^{{p}+1}}{{p}({p}-1)}\bigg [(\flat +1-\varsigma )^{p}(\flat -\varsigma +2+{p})-(\flat -\varsigma )^{p}(\flat -\varsigma +2+2{p})\bigg ]\bigg ]\\&-\frac{t_{\varsigma -1}^{{q}-1}\mathcal {K}_4\big ({A}(t_{\varsigma -1}),t_{\varsigma -1}\big )}{\Delta }\bigg [\frac{\Delta ^{{p}+1}}{{p}({p}-1)}\bigg [(\flat +1-\varsigma )^{{p}+1}-(\flat -\varsigma )^{p}(\flat -\varsigma +1+{p})\bigg ]\bigg ]\bigg ). \end{aligned}\right. \end{aligned}$$

47 $$\begin{aligned} {C}(t_{\flat +1})= & {} \left\{ \begin{aligned}&{C}_0+\frac{(1-{p})}{\mathcal {ABC}({p})}(t_{\flat +1}^{{q}-1})\bigg [\mathcal {K}_5\big ({C}(t_{\flat }),t_{\flat }\big )\bigg ]+\frac{{q}{p}}{\mathcal {ABC}({p})\Gamma ({p})}\sum _{\varsigma =0}^\flat \bigg (\frac{t_\varsigma ^{{q}-1}\mathcal {K}_5\big ({C}(t_{\varsigma }),t_{\varsigma }\big )}{\Delta }\\&\times \bigg [\frac{\Delta ^{{p}+1}}{{p}({p}-1)}\bigg [(\flat +1-\varsigma )^{p}(\flat -\varsigma +2+{p})-(\flat -\varsigma )^{p}(\flat -\varsigma +2+2{p})\bigg ]\bigg ]\\&-\frac{t_{\varsigma -1}^{{q}-1}\mathcal {K}_5\big ({C}(t_{\varsigma -1}),t_{\varsigma -1}\big )}{\Delta }\bigg [\frac{\Delta ^{{p}+1}}{{p}({p}-1)}\bigg [(\flat +1-\varsigma )^{{p}+1}-(\flat -\varsigma )^{p}(\flat -\varsigma +1+{p})\bigg ]\bigg ]\bigg ). \end{aligned}\right. \end{aligned}$$

## Parameter estimation

**Figure 1:**
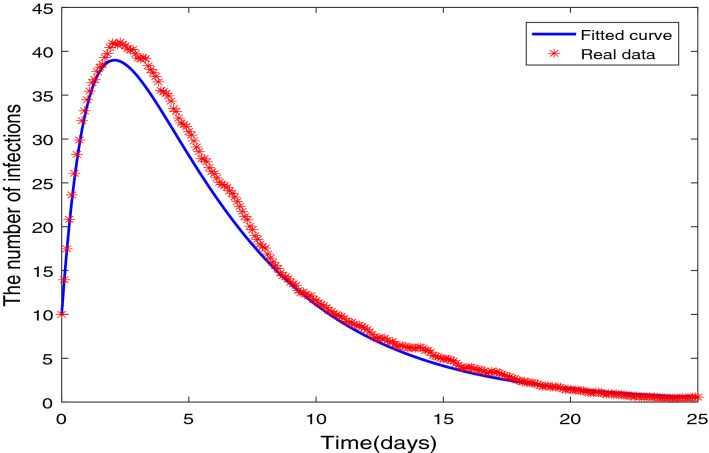
Quantity of the NoV infections compare with the numerical results of integer-order model (2).

In reference^34^, they study the data about a NoV infectious diarrhea incident reported in a middle school in a city. To guarantee the correctness and effectiveness of the methods, the data of a 2007 NoV outbreak in a middle school in one city is used as the real data to solve the inverse problem of the parameter estimation. For the daily reports and the data set in the Eq. (48), and the related relative error is used in the goodness of fit.

48 $$\begin{aligned} \min \left( \frac{\sum _{i=1}^{n}\left( A_{i}-{\hat{A}}_{i}\right) ^{2}}{\sum _{\iota =1}^{n} A_{\iota }^{2}}\right) , \end{aligned}$$

where $A_i$ is respectively the reported total number of infected, and ${\hat{A}}_i$ is the simulated total number of infected. The simulated cumulative number of infected are calculated by summing the individuals transit from the infected compartment to the recovered compartment for each day. Figure 1 shows the fit of model to the real data. Estimated values of parameters are shown in the Table 1.

**Table 1:** Biological parameters used in the proposed NoV model.

Parameter	Value	Source
$\Lambda$	120.0166	Estimated
$\rho$	6.6110	Fitted
$\eta$	$8.3452\times 10^{-11}$	Fitted
*d*	0.412	Fitted
$\delta$	0.333	Fitted
$\alpha$	0.3021	Fitted
$\tau$	0.90	Estimated

## Numerical simulations

In this section, we will illustrate our analytical results by some examples with the help of numerical simulations firstly. We use the first order stochastic iterative techniques of the fourth order Range kutta method to estimate the system solution (1). We adopt stochastic methodologies to simulate the double stochastic integrals because the system is driven by five independent noises $dW_i(t)$ for $i=1,2,3,4,5$. These integrals are approximated utilising the pseudo periodicity of the brownian bridge and their similarity to the deterministic Fourier series approach in our numerical method. For this, we use fourth-order Range kutta stochastic iterative techniques to accomplish the resulting discretization-transformation of the model (1),

49 $$\begin{aligned} H_{i+1}&=H_i+\bigg [\Lambda -\frac{\eta H_i(t) A_i(t)}{N}-(\rho +d) H_i(t)\bigg ]\Delta t+\zeta _{1} H_i(t) \sqrt{\Delta t}\xi _{1,i}+\frac{\zeta _{1}^2}{2}H_i(\xi _{1,i}^2-1)\Delta t\\ V_{i+1}&=V_i+\bigg [\rho H(t)-\frac{(1-\tau )\eta V(t)A(t)}{N}-d V(t)\bigg ]\Delta t+\zeta _{2} V_i(t) \sqrt{\Delta t}\xi _{2,i}+\frac{\zeta _{2}^2}{2}V_i(\xi _{2,i}^2-1)\Delta t\\ U_{i+1}&=U_i+\bigg [\frac{\eta H_i(t)A_i(t)}{N}+\frac{(1-\tau )\eta V_i(t)A_i(t)}{N}-(\alpha +d)U_i(t)\bigg ] \Delta t\\&\quad +\zeta _{3} U_i \sqrt{\Delta t}\xi _{3,i}+\frac{\zeta _{3}^2}{2}U_i(t)(\xi _{3,i}^2-1)\Delta t\\ A_{i+1}&=A_i+\bigg [\alpha U_i(t)-(\delta +d) A_i(t)\bigg ]\Delta t+\zeta _{4} A_i \sqrt{\Delta t}\xi _{4,i}+\frac{\zeta _{4}^2}{2}A_i(\xi _{4,i}^2-1)\Delta t\\ C_{i+1}&=C_i+\bigg [\delta A_i(t)-d C_i(t)\bigg ]\Delta t+\zeta _{4} C_i \sqrt{\Delta t}\xi _{4,i}+\frac{\zeta _{4}^2}{2}C_i(\xi _{5,i}^2-1)\Delta t \end{aligned}$$

Here $\xi _{k, i}(k=1,2,3,4)$ are four free Gaussian general variables with *N*(0, 1) and $\Delta t>0$ time-increment.

The values of the parameters listed in Table 2.

**Table 2:** The values for parameters given in model (1).

Parameters	Description	Set A	Set B	Set C
$\Lambda$	The recruitment rate	5.0	2.5	2.5
$\eta$	The effective contact rate	0.02	0.02	0.04
$\rho$	The vaccination coverage rate	0.03	0.01	0.05
*d*	The natural mortality rate	0.02	0.02	0.2
$\delta$	The recovery rate	0.05	0.5	0.4
$\alpha$	Developing clinical symptoms	0.02	0.2	0.3
$\tau$	The vaccine efficiency	1.0	0.90	0.90
$\zeta _1$	Noise intensity	0.2	0.7	00
$\zeta _2$	Noise intensity	0.5	0.9	00
$\zeta _3$	Noise intensity	0.6	0.7	00
$\zeta _4$	Noise intensity	0.2	0.14	00
$\zeta _5$	Noise intensity	0.6	0.10	00
*H*(0)	Initial value	75	75	75
*V*(0)	Initial value	20	20	20
*U*(0)	Initial value	55	55	55
*A*(0)	Initial value	30	30	30
*C*(0)	Initial value	20	20	20

### Numerical simulations for stochastic stability

Now, we will use numerical simulation to investigate the numerical approximation and biological feasibility of the system (1). As a result, we used the parameters and noise intensities value from Table 2. The initial value of the Individuals susceptible *H*(*t*), vaccinated *V*(0), asymptomatic *U*(0), symptomatic *A*(*t*), and recovered *R*(*t*) are presented in Table 2 for $t\in [0{-}150]$.

**Figure 2:**
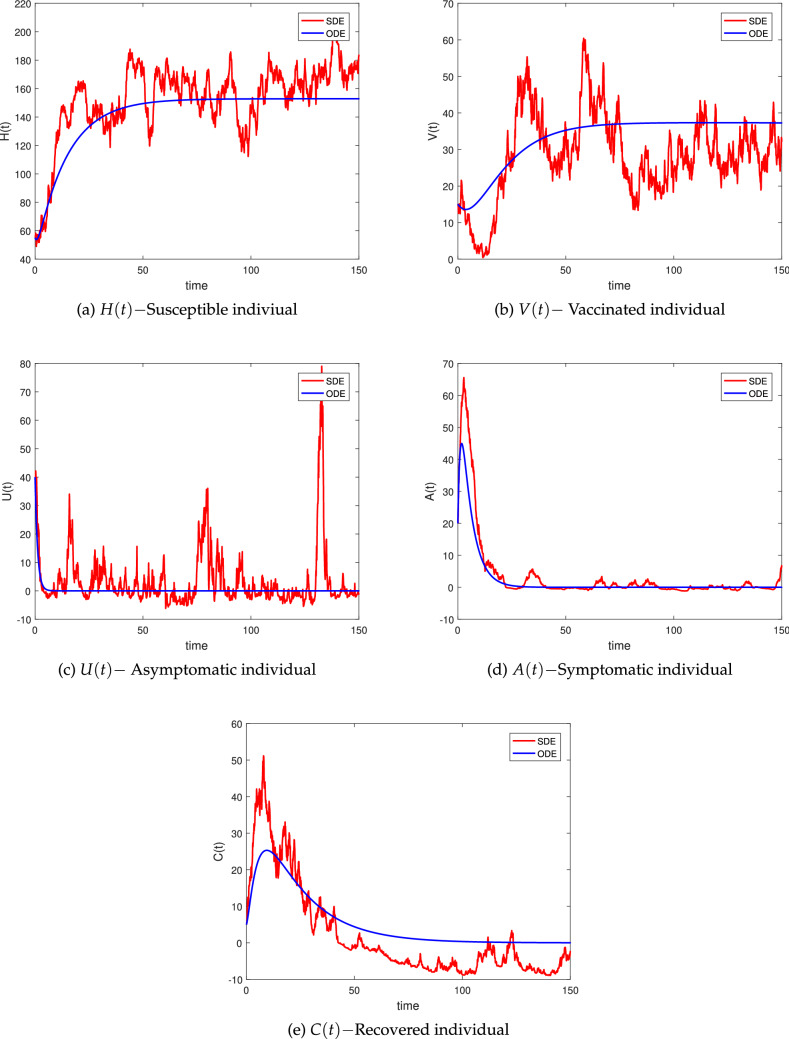
Simulations of (*H*(*t*), *V*(*t*), *U*(*t*), *A*(*t*), *C*(*t*)), for the stochastic models (1) with its corresponding deterministic version.

Taking into account white noises and parameter values from Table 2 (Set A), which ensure the conditions of Theorem 2, therefore, the infected population exponentially tends to zero with probability 1. The epidemic free equilibrium point shows global asymptotic stability in the related deterministic approach. As seen in Fig. 2, the disease can die.

**Figure 3:**
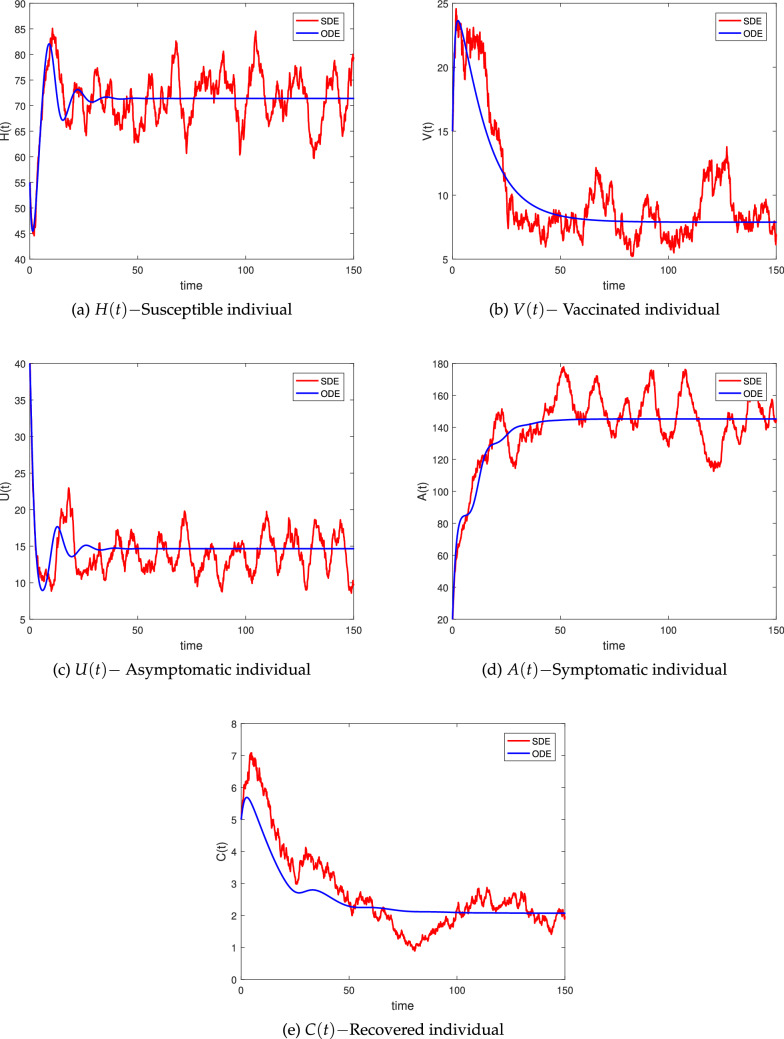
Simulations of (*H*(*t*), *V*(*t*), *U*(*t*), *A*(*t*), *C*(*t*)) for the stochastic models (1) with its corresponding deterministic version.

**Figure 4:**
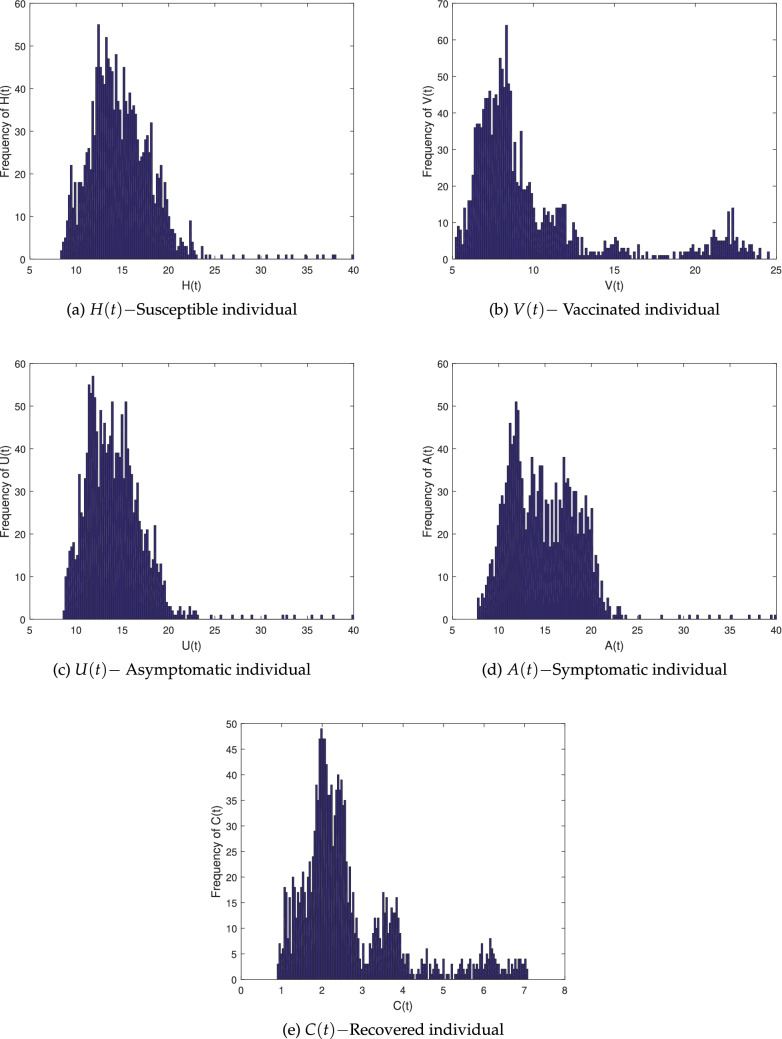
The probability distribution histogram of (*H*(*t*), *V*(*t*), *U*(*t*), *A*(*t*), *C*(*t*)) for the stochastic model (1).

We use parameter value from Table 2 (Set B) for the stochastic system (1) and compute $$ R^s_0>1$$, which ensure the condition for the NoV persistent. As shown in Fig. 3, the infection of system (1) will reside in the average, confirming the conclusions of Theorem 3. Observe 9000 attempts at $$t=500$$, then compute the average value. Theorem 3 suggests that the system (1) has an ergodic stationary distribution, as shown in Fig. 4.

#### Example 1

(Stochastic disease-free dynamical behavior) The parameter values are taken from Table 2 (Set A). As a result, we obtain the reproduction number ${\tilde{R}}^s_0<1$, and the root of the model (1) may be satisfied by Theorem 2.

$$\begin{aligned} \lim _{t\rightarrow \infty } \sup {\frac{\text {log} U(t)}{t}} \le < 0, \,\,\,\,\, a.s. \end{aligned}$$

and

$$\begin{aligned} \lim _{t\rightarrow \infty } \sup {\frac{\text {log} A(t)}{t}} \le < 0, \,\,\,\,\, a.s. \end{aligned}$$

As a result, the pandemic will disappear from the population, as seen in Fig. 2 demonstrates that the numerical-simulation validates our strategy.

#### Example 2

(Stochastic endemic dynamical behavior) We get the parameter values from Table 2 (Set B). We show that $R^s_0 >1$, and that the illness will lie or stabilised according to Theorem 3, and we illustrate our findings in Fig. 3. Theorem 3 states that the system (1) has just one stationary distribution, as shown in Fig. 3.

### Numerical simulation for fractal–fractional system

**Figure 5:**
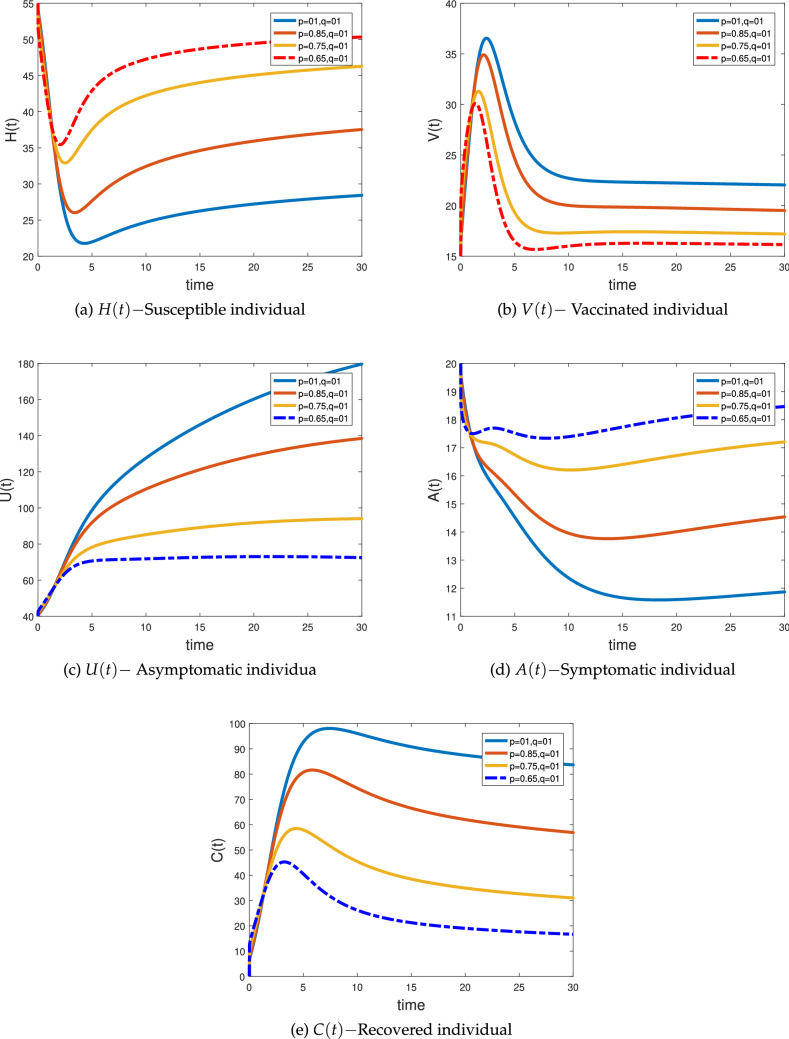
Simulations of the fractional-fractal NoV model in ABC case of system (36) when ${p}=(01, 0.85, 0.75, 0.65)$ and ${q}=(01, 01, 01, 01, 01)$.

**Figure 6:**
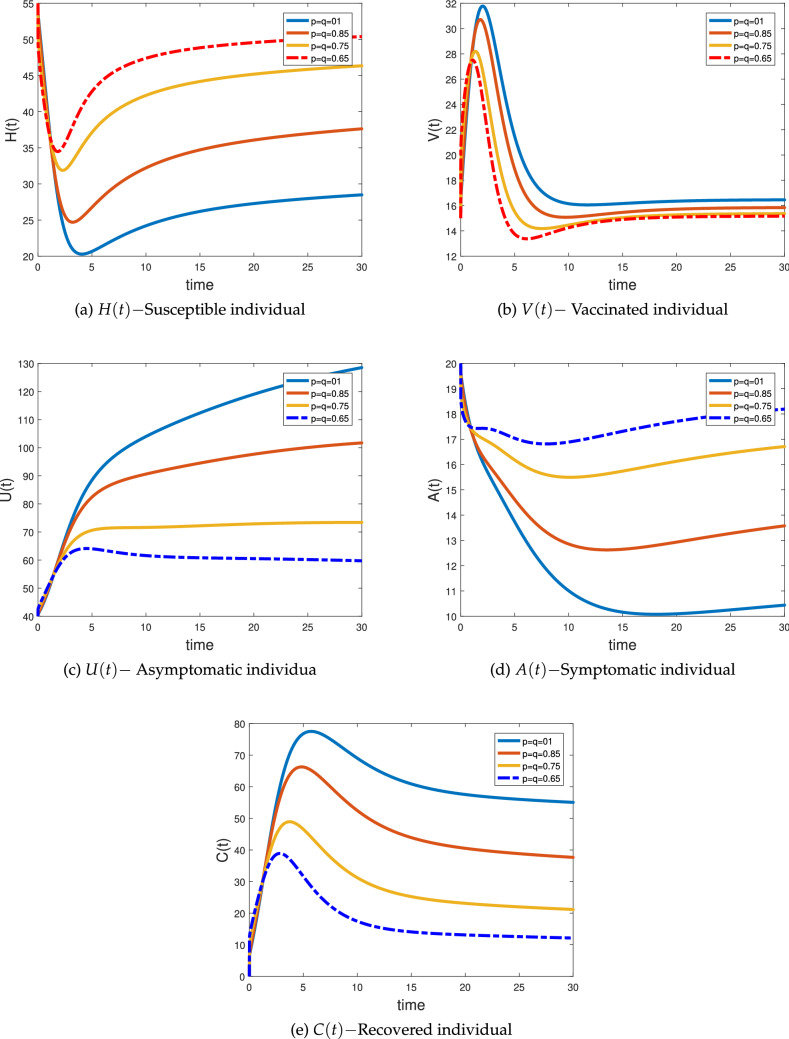
Simulations of the fractional-fractal NoV model in ABC case of system (36) when ${p}={q}=(01, 0.85, 0.75, 0.65)$.

In this section, we applied the novel numerical approach obtained above in (43)–(47) to simulate the proposed FF NoV model (36) having different arbitrary orders “${p}$” and various dimensions “${q}$” respectively given in Figs. 5 and 6, the initial and parameter value taken from Table 2 (Set C). We simulate the ABC fractal–fractional NoV model (36) when both the fractional order “${p}$” and fractal order “${q}$” are different. Figure 5a represents the susceptible class *H*(*t*) at different fractional orders and fractal dimensions. The class declines with the passage of time as the virus enters the society will transfer to the other classes of the system. This class converges quickly at low order and slowly at high orders. Figure 5b shows the vaccinated class *V*(*t*) growing at the beginning and then became stable at different fractal–fractional orders. As the disease control, the vaccination also controls and goes to their equilibrium point. Figure 5c is the representation of asymptotic or exposed class *U*(*t*) showing decrease in its behavior quickly with the passage of time like the behavior of susceptible class at different fractal–fractional orders. In Fig. 5 done can see the dynamical behavior of symptomatic or infectious class which also shows declines as controlled by vaccination and converging to their equilibrium point or became vanishes. Figure 5e shows the class of recovered population from the said epidemic at different fractional orders and fractal dimensions for the independent variable *t*. The cases of recovery increases at the beginning as more people have been vaccinated and the become stable. In Fig. 6 we simulate the ABC fractal–fractional NoV model (36) when both the fractional order “${p}$” and fractal order “${q}$” varies equally. From these figures, we note that changing both “${p}$” and “${q}$” at the same time an epidemic model provides interesting and biologically more feasible results because the infected population is vanishing more significantly as compared to the fractional systems. Thus, from these graphical results, we conclude that utilizing this new idea of FF operator one can observe more accurate results and provide deeper understanding not only for an epidemic model but also to the real world problem arising in science and engineering.

## Conclusion

In this paper, we propose an epidemic model for NoV transmission in considering environmental noise and fractal–fractional with vaccination effects. Based on the proposed model, there exist a unique time-global solution for any given positive initial value. The thresholds governing the extinction and propagation of the epidemic sickness are determined. The sufficient requirements for extinction of the disease and existence of ergodic stationary distribution of the stochastic system are then obtained from Theorems 2 and 3 utilizing Hasminskii theory and Lyapunov analysis methods. Finally, we applied the concept of FF calculus in the ABC sense to obtain the proposed model. In addition, numerical simulation are given to describe the solution behaviour of our theoretical result. We find that if the noise intensity is large, then the disease will go to extinction. The graphical simulations reveal that the fractal–fractional concept provides better and biologically more reliable results than the classical fractional and ordinary derivatives. In the future, the new approach of modeling known as fractal–fractional operator can be used confidently to study the dynamics of various infectious diseases including the novel COVID-19.

## Data Availability

Data sharing is not applicable to this article as no new data were created or analyzed in this study.
